# Silylated Sulfuric Acid: Preparation of a Tris(trimethylsilyl)oxosulfonium [(Me_3_Si−O)_3_SO]^+^ Salt

**DOI:** 10.1002/anie.202104733

**Published:** 2021-05-11

**Authors:** Kevin Bläsing, Rene Labbow, Axel Schulz, Alexander Villinger

**Affiliations:** ^1^ Institut für Chemie Universität Rostock Albert-Einstein-Strasse 3a 18059 Rostock Germany; ^2^ Leibniz-Institut für Katalyse e.V. Albert-Einstein-Strasse 29a 18059 Rostock Germany

**Keywords:** Lewis acid, silylium ion, structure, sulfate, synthesis

## Abstract

The chemistry of silylated sulfuric acid, O_2_S(OSiMe_3_)_2_ (T_2_SO_4_, T=Me_3_Si; also known as bis(trimethylsilyl) sulfate), has been studied in detail with the aim of synthesizing the formal autosilylation products of silylated sulfuric acid, [T_3_SO_4_]^+^ and [TSO_4_]^−^, in analogy to the known protonated species, [H_3_SO_4_]^+^ and [HSO_4_]^−^. The synthesis of the [TSO_4_]^−^ ion only succeeds when a base, such as OPMe_3_ that forms a weakly coordinating cation upon silylation, is reacted with T_2_SO_4_, resulting in the formation of [Me_3_POT]^+^[TSO_4_]^−^. [T_3_SO_4_]^+^ salts could be isolated starting from T_2_SO_4_ in the reaction with [T−H−T]^+^[B(C_6_F_5_)_4_]^−^ or T^+^[CHB_11_Br_6_H_5_]^−^ when a weakly coordinating anion is used as counterion. All silylated compounds could be crystallized and structurally characterized.

## Graphical abstract

The “larger” analog of protonated sulfuric acid, [H_3_SO_4_]^+^, the silylated oxosulfonium ion, [(Me_3_Si)_3_SO_4_]^+^, was obtained in the reaction of silylated sulfuric acid with a [Me_3_Si]^+^ transfer reagent such as [Me_3_Si−H−SiMe_3_]^+^[wca]^−^, when a weakly coordinating anion ([wca]^−^) is used as counterion.

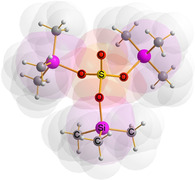

Almost 50 years of silylium ion chemistry have shown that many applications for silylium ions in the field of catalysis have emerged from the pure basic research of the first decades.[^1^, ^2^, ^3^, ^4^, ^5^, ^6^, ^7^, ^8^, ^9^] The development of silylium ion chemistry is closely related to carbenium ion chemistry, and it is no coincidence that silicon is also called the “kissing cousin” of carbon.^[2]^ And while we are on the subject of relationships: The [Me_3_Si]^+^ ion (T^+^) can also be understood as the “big brother” of the proton (Scheme 1).[^10^, ^11^, ^12^, ^13^, ^14^] Replacing H^+^ with T^+^ has several advantages. Substitution usually results in a thermodynamic (e.g. through hyperconjugation) and kinetic stabilization (through a larger steric demand) of the species under consideration. In the case of the pseudohalogen acids HX vs. TX (X=pseudohalogen),[^15^, ^16^, ^17^] for example, this leads to a significant stabilization, as can be seen in the increased melting and boiling points as well as the reluctance to oligomerize (e.g. X=CN, SCN, OCN). While HN_3_ is a highly explosive substance, TN_3_ can be handled safely even in large quantities at higher temperatures.[^18^, ^19^, ^20^, ^21^] Nevertheless, the chemistry of a protonated species is often similar to that of a silylated species (Scheme 1). For example, a classical neutralization reaction can also be formulated for the silylated species. Furthermore, the neutral dimers H_2_ and hexamethyldisilane T_2_, (Me_3_Si)_2_, show a similar reactivity towards dihalogens (X_2_), that is, they form HX and TX, respectively, in the reaction with X_2_, even with X=I.[^22^, ^23^] Like a free proton that does not exist in the condensed phase, also the T^+^ ion is always coordinated either to a neutral solvent, anion or any other Lewis basic site in a molecular system. Therefore, it is not surprising that in analogy to the protonated species, such as [H−X−H]^+^ (X=halogen,[^24^, ^25^] pseudohalogen),[^26^, ^27^] [H_*n*+1_E]^+^] (E=element of group 15[^28^, ^29^] for *n=*3 and 16[^28^, ^30^, ^31^, ^32^] for *n=*2) or arenium ions^[12]^ in aromatic systems, also the silylated species[^13^, ^14^, ^33^, ^34^, ^35^, ^36^] can be isolated in the presence of a weakly coordinating anion (Scheme 1).[^4^, ^6^] Like the protonated species, all these silylated cations should be regarded as strong Lewis acids that can be utilized as T^+^ transfer reagents.

**Scheme 1:**
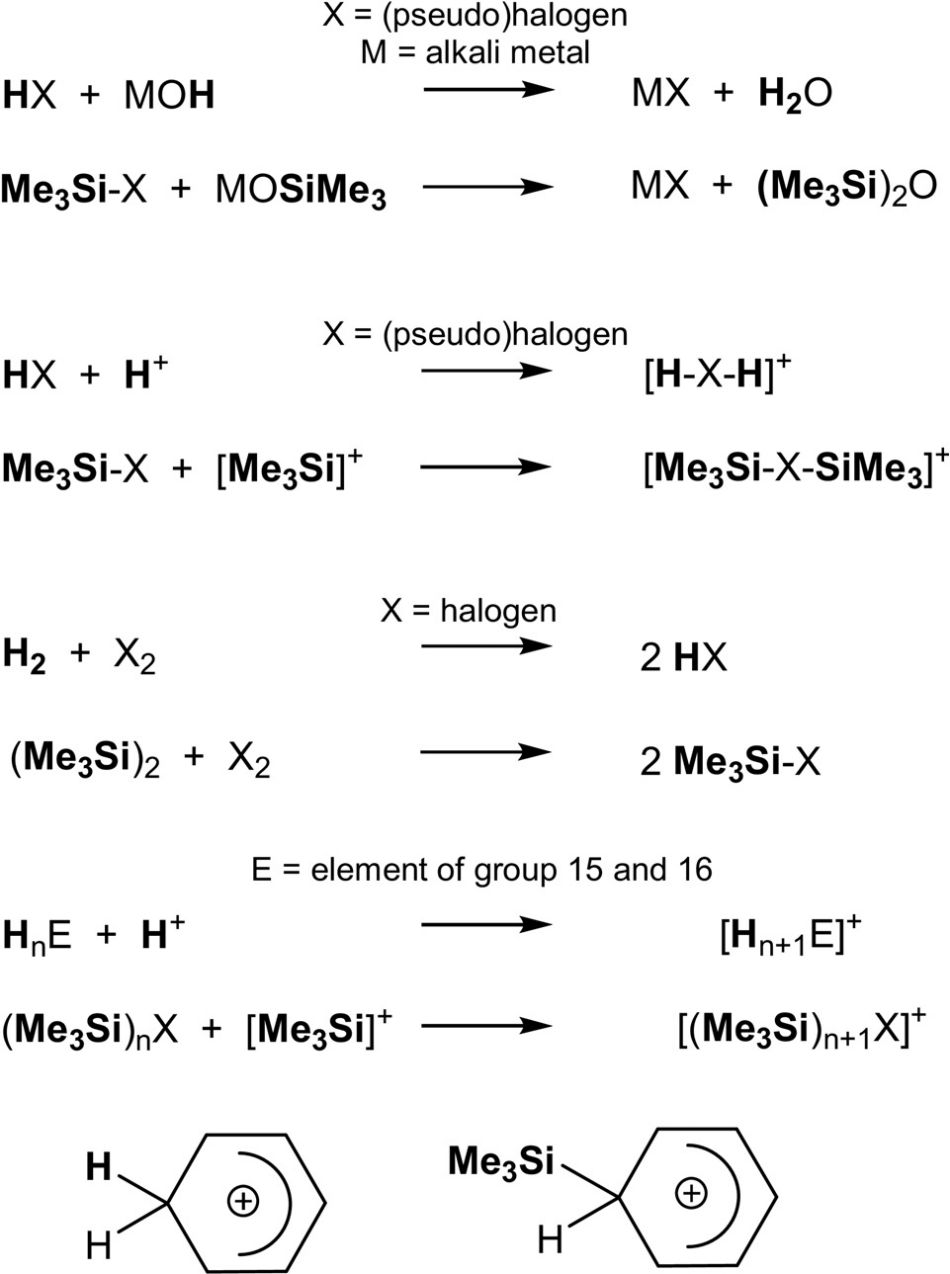
Similarities in the chemistry of an H^+^ and a [Me_3_Si]^+^ ion.

Interestingly, while the chemistry of T_3_PO_4_ and its silylated cationic species [T_4_PO_4_]^+^ has been explored,[^37^, ^38^, ^39^] nothing has been reported about a silylated cation of the type [T_3_SO_4_]^+^ to the best of our knowledge. However, protonated sulfuric acid, [H_3_SO_4_]^+^, was isolated by Minkwitz et al. in a super acidic system (HF/SbF_5_) as [SbF_6_]^−^ salt.^[40]^ As early as 1945, Patnode and Schmid reported on the synthesis of bis(trimethylsilyl)sulfate, T_2_SO_4_ (**1**), which they obtained in the reaction of TCl with H_2_SO_4_ (Scheme 2, Eq. 1).^[41]^ Since then, T_2_SO_4_ has often been used as a silylation reagent.[^42^, ^43^, ^44^, ^45^, ^46^, ^47^, ^48^, ^49^, ^50^] Following our interest in [Me_3_Si]^+^ chemistry, we studied the similarities between sulfuric acid, H_2_SO_4_, and its silylated congener T_2_SO_4_. Especially, we were intrigued by the idea to synthesize the formal autosilylation products of 2 T_2_SO_4_→[T_3_SO_4_]^+^+[TSO_4_]^−^ in analogy to the autoprotolysis reaction of sulfuric acid: 2 H_2_SO_4_→[H_3_SO_4_]^+^+[HSO_4_]^−^.

**Scheme 2:**
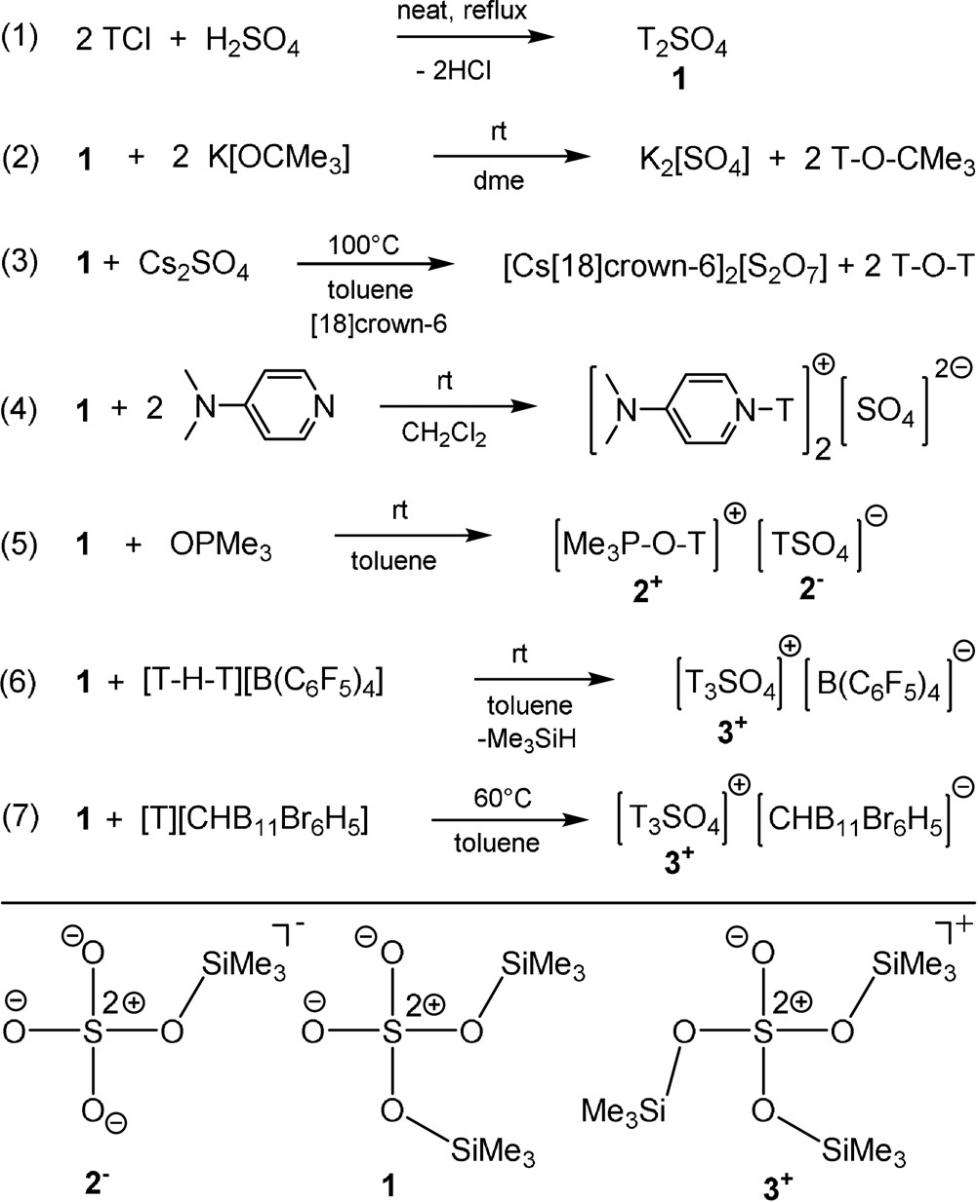
Synthesis of silylated sulfuric acid species T_2_SO_4_ (**1**), [TSO_4_]^−^ **(2^−^**) and [T_3_SO_4_]^+^ (**3^+^**). Bottom: Lewis representations of **1**, **2^−^** and **3^+^** (T=Me_3_Si).

We started this project with the synthesis of crystalline T_2_SO_4_ (**1**, T=Me_3_Si) from TCl and 95 % H_2_SO_4_ (Scheme 2, Eq. 1, Figure 1), which we obtained in 33 % yield after vacuum distillation at 100 °C (10^−3^ mbar, see SI). With T_2_SO_4_ in hand, we reacted it with various bases, such as DMAP (4‐(dimethylamino)pyridine), KO*t*Bu and OPMe_3_ to “neutralize” exactly one T^+^ ion in order to generate [TSO_4_]^−^ (Scheme 2, Eq. 2–5). With KO*t*Bu as base (independent of the stoichiometry), we always isolated K_2_SO_4_ and observed in solution the formation of the ether T−O−*t*Bu as evidenced by ^1^H, ^13^C and ^29^Si NMR studies. Also, the reaction of Cs_2_(SO_4_) with T_2_SO_4_ in toluene in the presence of [18]crown‐6 (to increase the solubility) did not lead to the formation of a [TSO_4_]^−^ salt, but the pyrosulfate [Cs[18]crown‐6]_2_S_2_O_7_ (X‐ray, see SI) and T−O−T (NMR) were produced in a condensation reaction. The reaction with DMAP was carried out in 2:1, 1:1 and 1:2 ratios in CH_2_Cl_2_ and followed by ^14^N, ^29^Si and ^17^O NMR spectroscopy (Figure S1a–c). In the ^14^N spectra, a strong broadening and shift of the two DMAP resonances (*δ*[^14^N]=−324.9 and −104.7 ppm) were observed, increasing with increasing amount of T_2_SO_4_. The two resonances (153 and 174 ppm) in the ^17^O NMR spectra are shifted to higher field with increasing amount of T_2_SO_4_ and the broad resonance at 153 ppm even vanishes. Interestingly, in the ^29^Si NMR studies, we always observed only one resonance strongly shifted and not resolved compared to that of pure T_2_SO_4_ (pure T_2_SO_4_: *δ*[^29^Si]=33.6 ppm, cf. T_2_SO_4_/DMAP ratio: 2:1 29.7, 1:1 27.7 and 1:2 24.4 ppm, Figure S1c). Therefore, we assume a highly dynamic DMAP/T_2_SO_4_ system from which we could only isolate crystalline [DMAP−T]_2_SO_4_ (X‐ray, see SI). To avoid the problems as discussed before, we tried the slightly weaker base OPMe_3_, which then, indeed, led to success. When exactly one equivalent of OPMe_3_ is reacted with one equivalent of pure T_2_SO_4_ in toluene, a crystalline trimethylsilylsulfate salt, [Me_3_P−O−T][TSO_4_], is obtained after concentration of the solution in 77 % yield (**2**, Scheme 2, Eq. 5, Figure 1). Only on one occasion could we isolate from such a reaction mixture one crystal of a side product, which was found to be the doubly desilylated pyrosulfate, [Me_3_PO−T]_2_[S_2_O_7_] (X‐ray, see SI).

**Figure 1:**
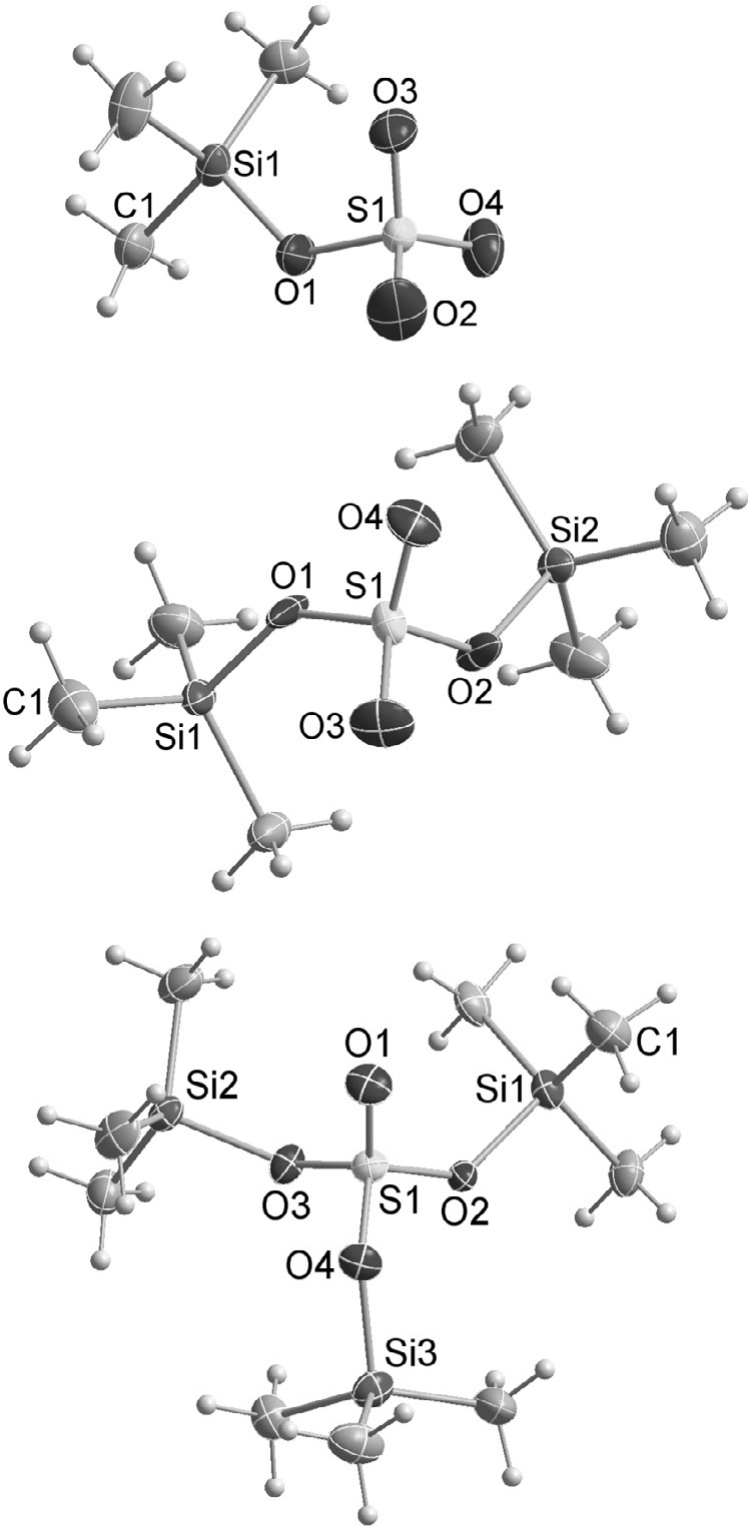
ORTEP representation of [TSO_4_]^−^ (**2^−^**, top), T_2_SO_4_(**1**, middle) and [T_3_SO_4_]^+^ (**3^+^**, bottom) in the crystal. The counterions of **2^−^** ([Me_3_POT]^+^) and **3^+^** ([CHB_11_Br_6_H_6_]^−^) are omitted for clarity (see SI). Ellipsoids are set at 50 % probability (123 K). Selected bond lengths and angles are listed in Table 1.

The synthesis of a tris(trimethylsilyl)oxosulfonium [T_3_SO_4_]^+^ salt is achieved by reacting [T−H−T][B(C_6_F_5_)_4_] with silylated sulfuric acid in an 1:1 ratio in toluene. Attempts to crystallize the salt [T_3_SO_4_][B(C_6_F_5_)_4_] failed both at room temperature and at lower temperatures such as 5 °C and −20 °C. Attempts to remove the entire solvent in vacuum (1–10^−3^ mbar) at 60 °C resulted in the decomposition of the salt, which can be observed by the formation of a black insoluble residue.^[12]^ The addition of non‐polar solvents such as *n*‐hexane to precipitate the salt also failed. Changing the solvent from toluene to 1,2‐dichlorobenzene was also unsuccessful. For this reason, we changed the counterion, as we assumed that the decomposition was initiated by a C−F activation at the borate anion. It is known that carborates are much more chemically robust compared to the [B(C_6_F_5_)_4_]^−^ anion.[^4^, ^12^, ^51^] Indeed, when [Me_3_Si][CHB_11_Br_6_H_5_] is reacted with T_2_SO_4_ in toluene, colorless crystals of the desired [T_3_SO_4_]^+^‐salt are obtained in 68 % yield after 30 min ultrasound treatment at 60 °C and recrystallization (Scheme 2, Eq. 7). The formation of the [T_3_SO_4_]^+^‐ion with [CHB_11_Br_6_H_5_]^−^ as counterion was unequivocally proven by single‐crystal X‐ray studies (Figure 1, bottom). It should be noted that although we were able to generate the formal autosilylation products of T_2_SO_4_ by separate synthesis routes, dissociation into [T_3_SO_4_]^+^ and [TSO_4_]^−^ was not observed for T_2_SO_4_, but [T_3_SO_4_]^+^ and [TSO_4_]^−^ react to give two T_2_SO_4_ molecules immediately.

All three silylated sulfuric acid species [TSO_4_]^−^, T_2_SO_4_ and [T_3_SO_4_]^+^ were studied by different ^13^C, ^17^O, ^29^Si, and ^31^P NMR techniques in solution (see SI) as well as IR/Raman spectroscopy. As expected, the ^29^Si resonance of [T_3_SO_4_]^+^ (*δ*[^29^Si]=54.1) was shifted by 22.2 ppm to lower field compared to T_2_SO_4_ (*δ*[^29^Si]=31.9), while a small high‐field shift by 3.9 ppm was observed for [TSO_4_]^−^ (*δ*[^29^Si]=28.0, cf. 32 [Me‐CN‐SiMe_3_]^+^,^[13]^ 35.6 [T_4_PO_4_]^+^,^[37]^ and computed 385 ppm for naked [Me_3_Si]^+^_(g)_,[^52^, ^53^] see SI, Table S3). As the ^29^Si NMR chemical shifts can be used as an indicator for the silylium ion character (and the deviation from planarity, see below),[^52^, ^53^, ^54^] that is, for the strength of the [Me_3_Si]^+^ interaction with the solvent T_2_SO_4_, it can be assumed that T_2_SO_4_ is a rather strong coordinating solvent utilizing the scale by Cremer et al. (−50 to 90 ppm, cf. 90–190 weakly coordinating, 200–370 weakly interacting, 370–385 noncoordinating solvents and 385 ppm gas phase).^[52]^

Crystals of all three silylated sulfuric acid species are moisture sensitive but thermally considerably stable with defined melting points (Table 1; T_2_SO_4_: 48, [Me_3_PO‐T][TSO_4_]: 120 °C, and [T_3_SO_4_][CHB_11_Br_6_H_6_]: 114 °C). Interestingly, while [T_3_SO_4_][B(C_6_F_5_)_4_] begins to decompose upon concentration in solution at ambient temperatures, [T_3_SO_4_][CHB_11_Br_6_H_6_] can be isolated in substance and even melts without decomposition, while decomposition occurs only above 160 °C.

**Table 1:** Selected bond lengths [Å] and angles [°],^[55]^ melting points [°C], NMR data [ppm], charge (transfer) [*e*], and trimethylsilyl affinities (TMSA) [kcal mol^−1^].

	[TSO_4_]^−^	T_2_SO_4_	[T_3_SO_4_]^+^
S−O^[a]^	1.422–1.437	1.399–1.466	1.410
S−O_(‐Si)_^[b]^	1.588	1.480–1.541	1.488–1.503
Si−O	1.683	1.731–1.738	1.761–1.782
O‐S‐O	110.6–114.9	118.1	–
O‐S‐O_(‐Si)_	104.2–107.5	103.3–110.7	111.3–114.8
O_(Si)_‐S‐O_(‐Si)_	–	103.7	104.7–106.4
Si‐O‐S	127.5	129.7–137.0	133.1–135.6
Σ∡Si	332.7	339.2–339.7	342.4–344.4
m.p.	120	48	114
*δ*[^29^Si]	28.0	31.9	54.1
*q*_(S)_	2.572	2.639	2.702
*q* ${}_{\left(\mathrm{SO}{}_{4}\right)}$ ^[c]^	−1.582	−1.350	−1.216
Δ*q* ^tot^_CT,T+_	0.418	0.650	0.784
TMSA^[d]^	215.9	82.9	55.5

[a] Corresponds to *d*(S−O) with O only bound to S. [b] Corresponds to *d*(S−O_(‐SiMe3)_) with O in a S−O−SiMe_3_ unit. [c] Cf. −2 in [SO_4_]^2−^, Δ*q* ^tot^_CT_=*q* ${}_{\left(\mathrm{SO}{}_{4}\right)}$ —(−2). [d] Trimethylsilyl affinity (TMSA) of A_(g)_ is defined as the negative of the reaction enthalpy Δ*H*_(g)_° in kcal mol^−1^ at 298.15 K for the reaction A_(g)_+T^+^_(g)_→[AT]^+^_(g)_, that is the TMSA values given is for the conjugated acid–base pair A_(g)_/[AT]^+^_(g)_.

Crystallization of all three silylated sulfuric acid species from either *n*‐pentane (T_2_SO_4_) or toluene ([Me_3_PO−T][TSO_4_] and [T_3_SO_4_][CHB_11_Br_6_H_6_]) yielded colorless crystals (Figure 1). T_2_SO_4_ crystallized in the monoclinic space group *C*2/*c*, while [Me_3_POT][TSO_4_] and [T_3_SO_4_][CHB_11_Br_6_H_6_] crystallized in the orthorhombic space group *Pbca* and *P*2_1_2_1_2_1_, respectively. For all three compounds, there are only relatively weak intermolecular O⋅⋅⋅H−C interactions (Figures S2–S4, SI), but these are found in each case for the non‐silylated O atom of the SO_4_ core within the silylated species. That is, for [TSO_4_]^−^ with three non‐silylated O atoms one finds such interactions with three neighboring [Me_3_POT]^+^ cations (Figure S3), for T_2_SO_4_ correspondingly with two neighboring T_2_SO_4_ molecules (Figure S2) and in [T_3_SO_4_]^+^ exactly one such interaction (Figure S4), however, with one adjacent cation. In the latter case, interestingly, weak Br_anion_⋅⋅⋅H−C_cation_ interactions are added. Likewise, weak Br_anion_⋅⋅⋅H−C_anion_ interactions are found between the H atom attached to the C atom of one carborate anion and the Br atom in *para*‐position to the C−H bond atom of an adjacent second carborate anion (Figure S4). This leads to a zig–zag chain of carborate anions in the solid. The [T_3_SO_4_]^+^ cations coordinate with this chain via the above‐mentioned weak Br_anion_⋅⋅⋅H−C_cation_ interactions.

As depicted in Figure 1, the central SO_4_ core always adopts a highly distorted tetrahedral geometry, with two different S−O bond lengths (Table 1). In accord with electrostatic consideration, with increasing number of Me_3_Si groups, the Si−O bond lengths are elongated along [TSO_4_]^−^<T_2_SO_4_<[T_3_SO_4_]^+^. Similarly, the Si‐O‐S angles (127.5 to 134.2° (averaged)) and the sum of the angles around the Si atoms (from 332 to 344°) increase, indicating the largest silylium ion character in [T_3_SO_4_]^+^>T_2_SO_4_>[TSO_4_]^−^.

To get some insight into the charge transfer upon silylation and desilylation, respectively, we computed the partial net charges of the elements and the [Me_3_Si] as well as [SO_4_] moieties within all three silylated species (Table 1 and S6) at the pbe1pbe/aug‐cc‐pwCVDZ level of theory. Two interesting features can be derived from these data: (i) The atomic charges do not change much upon increasing silylation degree. For example, the partial charge at the central S atom only slightly increases along [SO_4_]^2−^ (2.572)<[TSO_4_]^−^ (2.589)<T_2_SO_4_ (2.639)<[T_3_SO_4_]^+^ (2.702 *e*) although the overall charge changes by Δ*q*=3 *e*. This moderate change in the atomic charges of sulfur can be attributed to delocalization over the entire molecular entity. (ii) The formal charge transfer per Me_3_Si group decreases along 0.418 [TSO_4_]^−^>0.325 T_2_SO_4_>0.26 *e* [T_3_SO_4_]^+^, while the total charge transfer increases in this direction (0.418<0.650<0.784 *e*). That is, the total charge of the SO_4_ core is most strongly reduced from −2 in [SO_4_]^2−^ to −1.216 *e* in [T_3_SO_4_]^+^. Finally, the trimethysilyl affinities (TMSA, Table 1) of all three silylated species were computed increasing along 55.5 ([T_3_SO_4_]^+^)<82.9 (T_2_SO_4_)<215.9 kcal mol^−1^ ([SO_4_]^2−^, cf. 32.8 [T−H−T]^+^, 76.6 [DMAP−T]^+^, 72.8 [Me_3_PO−T]^+^). Hence, [T_3_SO_4_]^+^ represents the best silylating species and most “naked” [Me_3_Si]^+^ species amongst the considered species here. Nevertheless within the [T−H−T]^+^ ion, “[Me_3_Si]^+^” is less strongly bound and can be used to generate [T_3_SO_4_]^+^ salts as experimentally demonstrated here and in accord with the computed TMSA value.

In summary, highly labile salts containing [T_3_SO_4_]^+^ and [TSO_4_]^−^ ions were generated using super‐Lewis acidic media and bulky, chemically robust counterions, similar to the chemistry known for analogous protonated species. Formally, [T_3_SO_4_]^+^ and [TSO_4_]^−^ ions can be viewed as the product of autosilylation of T_2_SO_4_ and the big proton analogs of [H_3_SO_4_]^+^ (protonated sulfuric acid) and [HSO_4_]^−^ (hydrogen sulfate).

## Conflict of interest

The authors declare no conflict of interest.

## Supporting information

As a service to our authors and readers, this journal provides supporting information supplied by the authors. Such materials are peer reviewed and may be re‐organized for online delivery, but are not copy‐edited or typeset. Technical support issues arising from supporting information (other than missing files) should be addressed to the authors.

Supplementary
