# Supplementary material for: Silylated Sulfuric Acid: Preparation of a Tris(trimethylsilyl)oxosulfonium [(Me3Si−O)3SO]+ Salt
Source: Angew Chem Int Ed Engl. 2021 May 11;60(25):13798–802. doi: 10.1002/anie.202104733 (PMC8251596; doi:10.1002/anie.202104733)
Supplement: Supplementary file 1 — Supplementary [file ANIE-60-13798-s001.pdf]

Angewandte Chemie
Eine Zeitschrift der Gesellschaft Deutscher Chemiker

Supporting Information

Silylated Sulfuric Acid: Preparation of a Tris(trimethylsilyl)oxosulfonium $[(Me_3Si{-}O)_3SO]^+$ Salt

Kevin Bläsing, Rene Labbow, Axel Schulz, and Alexander Villinger*

anie_202104733_sm_miscellaneous_information.pdf

Supporting Information

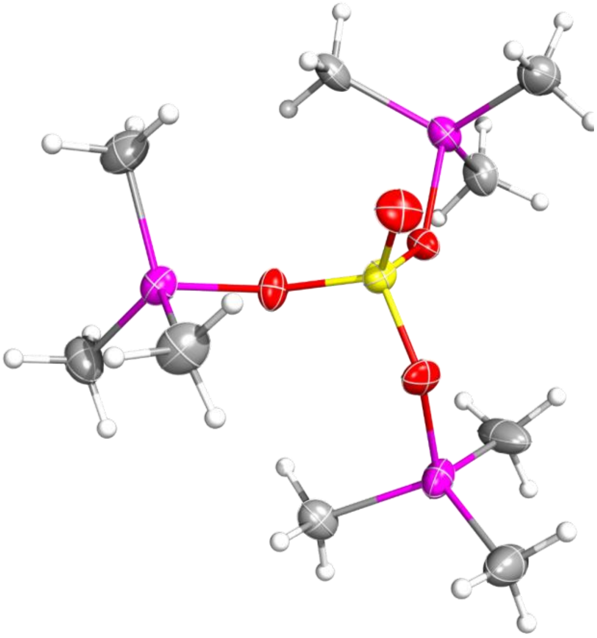

This file includes:

1 Experimental ... 3

2 Syntheses of starting materials ... 6

2.1 Bis(trimethylsilyl)sulfate – $(Me_3SiO)_2SO_2$ ... 6

2.2 4-*N*,*N*-(dimethylamino)pyridine – 4-DMAP ... 8

2.3 Sulfuric acid – H_2SO_4 (*conc.*) ... 8

2.4 Ammonium sulfate – $[NH_4]_2[SO_4]$ ... 9

2.5 Potassium *tert*-butoxide – $K[OCMe_3]$ ... 9

2.6 1,4,7,10,13,16-Hexaoxa-*cyclo*-octadecane – [18]crown-6 ... 10

2.7 Trimethylphosphinoxid – $OPMe_3$ ... 10

3 Syntheses of compounds ... 11

3.1 Synthesis and characterization of Bis-1-*N*-trimethylsilyl-4-*N'*,*N'*-dimethylamino-pyridinium sulfate ... 11

3.2 Synthesis and characterization of trimethylsiloxytrimethylphosphonium trimethylsilylsulfate – $[Me_3SiOPMe_3][(Me_3SiO)SO_3]$ 16

3.3 Synthesis and characterization of potassium sulfate – K_2SO_4 17

3.4 Synthesis and NMR characterization of tris(trimethylsiloxy)oxosulfonium tetrakis(pentafluoro)phenylborat: $[(Me_3SiO)_3SO][B(C_6F_5)_4]$ 18

3.5 Synthesis and NMR characterization of tris(trimethylsiloxy)oxosulfonium hexabromo pentahydro undecaborat: $[(Me_3SiO)_3SO][CHB_{11}Br_6H_5]$ 20

4 Structure elucidation 21

5 Comparison of experimental spectroscopic data 30

5.1 Comparison of NMR data 30

5.2 Comparison of structural data 30

6 Computational Details 31

7 References 49

1 Experimental

General Information. If not stated otherwise, all manipulations were carried out under oxygen- and moisture-free conditions under an inert atmosphere of argon using standard Schlenk or Drybox techniques. Solvents and reactants were obtained from commercial sources or synthesized as detailed in Table S1.

Table S1: Origin and purification of solvents and reactants.

Substance	Origin	Purification[1]
$C_6H_5CH_3$, DME, THF	local trade	purified according to literature procedure<br>dried over Na/benzophenone<br>freshly distilled prior to use
n-pentane, *n*-hexane	local trade	dried over Na/benzophenone/tetraglyme<br>freshly distilled prior to use
CH_3CN	local trade	dried over CaH_2 freshly distilled prior to use
1,2-DCB, CH_2Cl_2	local trade	dried over P_4O_{10} and CaH_2<br>freshly distilled and degassed (freeze-pump-thaw)
Li	Merck, ≥ 99 %	used as received
S_8	VEB Laborchemie Apolda	used as received
H_2SO_4	Chemsolute, 95 % p.A.	used as received
$OPMe_3$	Riedel-de-Haen AG, 98 %	recrystallized from CH_2Cl_2
K[$OCMe_3$]	Fluka, ≥ 97 %	sublimed *in vacuo* ($1\cdot10^{-3}$ mbar) at 220 °C
[18]crown-6	Fluka, 99 %	recrystallized twice from CH_3CN and THF
4-DMAP		recrystallized from toluene
Me_3SiCl	Merck	dried over CaH_2<br>freshly distilled prior to use
$(Me_3Si)_2S$	synthesized[2]	
$(Me_3SiO)_2SiO_2$	synthesized[3]	
$[Me_3Si)_2H][B(C_6F_5)_4]$	synthesized[4,5]	
$[Me_3Si][CHB_{11}Br_6H_5]$	synthesized[6–10]	
CD_2Cl_2	Euriso-Top, 99.5 %	dried over P_4O_{10} and CaH_2<br>freshly distilled prior to use
C_6D_6, $C_6D_5CD_3$	Euriso-Top, 99.5 %	dried over Na<br>freshly distilled prior to use

Substance	Origin	Purification[1]
THF-d_8	Euriso-Top, 99.5 %	dried over Na<br>distilled and stored over molecular sieves (4 Å)
Acetone-d_6	Euriso-Top, 99.5 %	stored over molecular sieves (4 Å)
DMSO-d_6	Euriso-Top, 99.5 %	dried over CaH_2<br>freshly distilled prior to use<br>stored over molecular sieves (4 Å)
Sodium piece	Aldrich, 99 %	
Benzophenon	Aldrich, 99 %	
Tetraglyme	Alfa Aesar, ≥ 95 %	

NMR spectra were recorded on Bruker spectrometers (AVANCE 250, AVANCE 300 or AVANCE 500) and were referenced internally to the deuterated solvent ($^{13}C\{^{1}H\}$: CD_2Cl_2 δ_{ref} = 54.0 ppm, acetone-d_6 δ_{ref} = 29.84 ppm, DMSO-d_6 δ_{ref} = 39.52 ppm, C_6D_6 δ_{ref} = 128.4 ppm, $C_6D_5CD_3$ δ_{ref} = 20.4 ppm, THF-d_8 $\delta_{ref,1}$ = 25.4 ppm, $\delta_{ref,2}$ = 67.6 ppm), to protic impurities in the deuterated solvent (^{1}H: $CDHCl_2$ δ_{ref} = 5.32 ppm, acetone-d_5 δ_{ref} = 1.72 ppm, DMSO-d_5 δ_{ref} = 2.50 ppm, C_6HD_5 δ_{ref} = 7.16 ppm, $C_6D_5CHD_2$ δ_{ref} = 2.08 ppm, THF-d_7 $\delta_{ref,1}$ = 1.73 ppm, $\delta_{ref,2}$ = 3.58 ppm) or externally ($^{14}N\{^{1}H\}$: CH_3NO_2 δ_{ref} = 0 ppm, ^{15}N: CH_3NO_2 δ_{ref} = 0 ppm, ^{17}O: H_2O δ_{ref} = 0 ppm, ^{29}Si: Me_4Si δ_{ref} = 0 ppm, ^{31}P: 85 % H_3PO_4 δ_{ref} = 0 ppm). All measurements were carried at ambient temperature unless stated otherwise. ^{15}N NMR shifts were derived from ^{1}H-^{15}N HMBC NMR spectra. NMR signals were assigned using experimental data (e.g. chemical shifts, coupling constants, integrals where applicable) in conjunction with computed NMR data (GIAO method, cf. Computational details, p. 31).

IR spectra of crystalline samples were recorded on a Bruker Alpha II FT-IR spectrometer equipped with an ATR unit at ambient temperature under argon atmosphere.

Raman spectra of crystalline samples were recorded using a LabRAM HR 800 Horiba Jobin YVON Raman spectrometer equipped with an Olympus BX41 microscope with variable lenses. The samples were excited by a red laser (633 nm, 17 mW, air-cooled HeNe laser), blue laser (473 nm, 20 mW, air-cooled solid-state laser). All measurements were carried out at ambient temperature unless stated otherwise.

Elemental analyses were obtained using an Elementar vario Micro cube CHNS analyser.

Melting points (uncorrected) were determined using a Stanford Research Systems EZ Melt at a heating rate of 5 °C/min.

DSC analyses were carried out at a heating rate of 5 °C/min using a Mettler-Toledo DSC 823e.

Mass spectra were recorded on a Thermo Electron MAT 95-XP sector field mass spectrometer using crystalline samples.

2 Syntheses of starting materials

2.1 Bis(trimethylsilyl)sulfate – $(Me_3SiO)_2SO_2$

$$Me_3SiCl + H_2SO_4 \xrightarrow[\text{-HCl}]{\text{neat, reflux}} (Me_3Si)_2SO_4$$

$(Me_3SiO)_2SO_2$ is synthesized according to a slightly modified literature procedure.[3]

To a stirred solution of trimethylsilyl chloride Me_3SiCl (32.5 g, 0.3 mol) concentrated sulfuric acid H_2SO_4 (95 %, ~14 g) is added dropwise about 15 minutes. The evolved hydrogen chloride was ducted through a bath of sodium hydroxide. At first the solution slightly cools down and appeared as slurry. After complete addition of the acid the reaction mixture becomes clear again. After an hour of additional stirring no further hydrogen chloride evolved. The solution is distilled *in vacuo* ($1{\cdot}10^{-3}$ mbar) at 110 °C (oil bath) resulting in (10.4 g, 0.05 mol, 33 %) colourless crystals of bis(trimethylsilyl)sulfate $(Me_3SiO)_2SO_2$ (**1**). Crystals suitable for X-ray crystallographic analysis were obtained by cooling 5 °C (refrigerator) a saturated *n*-pentane solution overnight.

$C_6H_{18}O_4SSi_3$ (214.36 g/mol): **mp**. 48 °C. **EA** calc. (found), %: C, 33.62 (33.72); H, 8.46 (8.26); S, 14.96 (15.31). **^{1}H NMR** (25 °C, toluene-d_8, 300.13 MHz): δ = 0.18 (s, SiCH_3, $^1J(^1H\text{-}^{13}C)$ = 120.7 Hz, $^2J(^1H\text{-}^{29}Si)$ = 7.1 Hz). **^{1}H NMR** (25 °C, C_6D_6, 300.13 MHz): δ = 0.18 (s, SiCH_3, $^1J(^1H\text{-}^{13}C)$ = 120.5 Hz, $^2J(^1H\text{-}^{29}Si)$ = 7.0 Hz). **^{1}H NMR** (25 °C, DMSO-d_6, 300.13 MHz): δ = 0.25 (s, SiCH_3, $^1J(^1H\text{-}^{13}C)$ = 120.1 Hz, $^2J(^1H\text{-}^{29}Si)$ = 7.0 Hz). **^{1}H NMR** (25 °C, CD_2Cl_2, 300.13 MHz): δ = 0.35 (s, SiCH_3, $^1J(^1H\text{-}^{13}C)$ = 120.5 Hz, $^2J(^1H\text{-}^{29}Si)$ = 6.7 Hz). **^{1}H NMR** (25 °C, 1,2-DCB, ref. ext. toluene-d_8, 300.13 MHz): δ = 0.22 (s, SiCH_3, $^1J(^1H\text{-}^{13}C)$ = 120.7 Hz), 6.85 (m, *m*-$H^{[1,2\text{-}DCB]}$), 7.11 (m, *o*-$H^{[1,2\text{-}DCB]}$). **^{13}C{^{1}H} NMR** (25 °C, toluene-d_8, 75.48 MHz): δ = –0.36 (s, SiCH_3, $^1J(^{13}C\text{-}^{29}Si)$ = 60.2 Hz). **^{13}C{^{1}H} NMR** (25 °C, C_6D_6, 75.48 MHz): δ = –0.30 (s, SiCH_3). **^{13}C{^{1}H} NMR** (25 °C, DMSO-d_6, 75.48 MHz): δ = 0.31 (s, SiCH_3, $^1J(^{13}C\text{-}^{29}Si)$ = 59.3 Hz). **^{13}C{^{1}H} NMR**

(25 °C, CD_2Cl_2, 75.48 MHz): δ = 0.16 (s, SiCH_3, $^1J(^{13}C-^{29}Si)$ = 60.0 Hz). **$^{13}C\{^1H\}$ NMR** (25 °C, 1,2-DCB, ref. ext. -toluene-d_8, 75.48 MHz): δ = –0.31 (s, SiCH_3, $^1J(^{13}C-^{29}Si)$ = 60.1 Hz), 127.86 (*m*-$CH^{[1,2\text{-DCB}]}$), 130.53 (*o*-$CH^{[1,2\text{-DCB}]}$), 132.49 (*ipso*-$CCl^{[1,2\text{-DCB}]}$). **^{17}O NMR** (25 °C, toluene-d_8, 67.83 MHz): δ = 152.61 (b, 2O, *O*SiMe$_3$, $\Delta\nu_{1/2}$ = 180 Hz), 174.49 (b, 2O, *O*S, $\Delta\nu_{1/2}$ = 75 Hz). **^{29}Si INEPT NMR** (25 °C, toluene-d_8, 59.52 MHz) δ = 31.84 (dec, *Si*CH_3, $^2J(^{29}Si-^1H)$ = 7.1 Hz). **^{29}Si INEPT NMR** (25 °C, C_6D_6, 59.52 MHz) δ = 31.93 (dec, *Si*CH_3, $^2J(^{29}Si-^1H)$ = 7.0 Hz). **^{29}Si INEPT NMR** (25 °C, DMSO-d_6, 59.52 MHz) δ = 28.51 (dec, *Si*CH_3, $^2J(^{29}Si-^1H)$ = 7.0 Hz). **^{29}Si INEPT NMR** (25 °C, CD_2Cl_2, 59.52 MHz) δ = 33.58 (dec, *Si*CH_3, $^2J(^{29}Si-^1H)$ = 6.7 Hz). **^{29}Si INEPT NMR** (25 °C, 1,2-DCB, 59.52 MHz) δ = 32.55 (dec, *Si*CH_3, $^2J(^{29}Si-^1H)$ = 7.2 Hz). **IR** (ATR, 8 scans, 25 °C, cm^{-1}): $\tilde{\nu}$ = 2968 (w), 2908 (w), 1465 (w), 1415 (w), 1351 (m), 1257 (m), 1187 (m), 1054 (w), 948 (s), 817 (s), 763 (s), 700 (m), 619 (m), 578 (m), 559 (m). **Raman** (473 nm, 6 mW, 30 s, 10 acc, 25 °C, cm^{-1}, 300 s photobleach): $\tilde{\nu}$ = 2978 (6), 2908 (10), 1420 (1), 1388 (1), 1350 (1), 1255 (1), 1185 (2), 980 (1), 857 (1), 770 (1), 705 (1), 664 (1), 614 (6), 531 (1), 364 (1), 252 (1), 233 (1), 195 (2). **MS** (CI^+, m/z (%)): 243 (8) $[(Me_3SiO)_2SO_2 + H]^+$, 229 (20) $[Me_3Si\text{-}SO_4\text{-}SiMe_2H + H]^+$, 171 (100) $[Me_3Si\text{-}SO_3H_2]^+$, 123 (77) $[HSiSO_3]^+$, 99 (77) $[H_3SO_4]^+$, 91 (17) $[Me_3SiOH_2]^+$, 79 (14) $[MeSO_2]^+$, 69 (17) $[(CH_2)_3Si]^+$, 61 (23) $[MeSiOH_2]^+$.

2.2 4-*N*,*N*-(dimethylamino)pyridine – 4-DMAP

$C_7H_{10}N_2$ (122.17 g/mol): **mp**. 113 °C. **^{1}H NMR** (25 °C, CD_2Cl_2, 300.13 MHz): δ = 2.98 (s, 6H, NC*H*$_3$, $^1J(^1H-^{13}C)$ = 136.3 Hz), 6.48 (m, 2H, *m*-C*H*), 8.15 (m, 2H, *o*-C*H*). **^{13}C{^{1}H} NMR** (25 °C, CD_2Cl_2, 75.48 MHz): δ = 39.35 (s, N*C*H$_3$), 107.07 (s, *m*-*C*H), 150.28 (s, *o*-*C*H), 154.79 (s, *ipso*-*C*). **^{14}N NMR** (25 °C, CD_2Cl_2, 36.14 MHz): δ = –324.88 (*N*CH$_3$, $\Delta\nu_{1/2}$ = 700 Hz), –104.66 (*N*(CH)$_2$, $\Delta\nu_{1/2}$ = 550 Hz). **IR** (ATR, 64 scans, 25 °C, cm^{-1}): $\tilde{\nu}$ = 3091 (w), 3031 (w), 3014 (w), 2995 (w), 2900 (m), 2867 (w), 2823 (m), 2807 (w), 2786 (w), 2750 (w), 1959 (w), 1926 (w), 1893 (w), 1594 (m), 1537 (m), 1513 (m), 1444 (m), 1436 (m), 1417 (m), 1375 (m), 1344 (m), 1276 (m), 1220 (s), 1137 (m), 1103 (m), 1068 (m), 985 (m), 943 (m), 804 (s), 750 (m), 661 (m), 538 (m), 528 (s). **Raman** (633 nm, 12 mW, 20 s, 10 acc., 25 °C, cm^{-1}): $\tilde{\nu}$ = 3083 (1), 3034 (1), 2999 (1), 2948 (1), 2912 (1), 2870 (1), 2856 (1), 2809 (1), 1613 (1), 1590 (1), 1523 (1), 1478 (1), 1464 (1), 1446 (1), 1416 (1), 1376 (1), 1348 (1), 1277 (1), 1230 (1), 1183 (1), 1123 (1), 1106 (1), 1063 (2), 984 (6), 948 (2), 825 (1), 806 (1), 748 (6), 663 (1), 531 (1), 480 (1), 400 (1), 262 (1), 119 (2), 74 (10), 62 (9).

2.3 Sulfuric acid – H_2SO_4 (*conc.*)

95% H_2O_4S: **^{17}O NMR** (25 °C, neat, ref. ext. to D_2O, 67.83 MHz): δ = 17.66 ($H_3O_x^+$, $\Delta\nu_{1/2}$ = 450 Hz), 151.88 (S*O*$_4$, $\Delta\nu_{1/2}$ = 750 Hz). **Raman** (473 nm, 6 mW, 10 s, 10 acc., 25 °C, cm^{-1}): $\tilde{\nu}$ = 3532-2632 (very broad), 1371 (1), 1149 (4), 1043 (3), 909 (10), 553 (4), 413 (2), 391 (2).

2.4 Ammonium sulfate – $[NH_4]_2[SO_4]$

H_4NO_4S (114.10 g/mol): **mp**. 344 °C (dec.). **IR** (ATR, 32 scans, 25 °C, cm^{-1}): 3369−2644 (very broad), 1402 (m), 1064 (s), 609 (s). **Raman** (632 nm, 6 mW, 15 s, 10 acc., 25 °C, cm^{-1}): $\tilde{\nu}$ = 3786-2680 (very broad), 1672 (1), 1444 (1), 1397 (1), 1227 (1), 1099 (1), 1060 (1), 971 (10), 608 (1), 447 (1), 165 (1).

NMR spectra was recorded as [18]crown-6 complex in D_2O.

^{1}H NMR (25 °C, D_2O, 250.13 MHz): δ = 3.73 (s, $OCH_2^{[18]crown-6}$, $^{1}J(^{1}H-^{13}C)$ = 143.3 Hz), 6.99 (b, NH_4, $\Delta\nu_{1/2}$ = 36 Hz). **^{13}C{^{1}H} NMR** (25 °C, D_2O ref. ext., 62.90 MHz): δ = 69.57 (s, $OCH_2^{[18]crown-6}$). **^{14}N NMR** (25 °C, D_2O, 36.14 MHz): δ = –359.69 (NH_4, $\Delta\nu_{1/2}$ = 5 Hz). **^{17}O NMR** (25 °C, D_2O, 59.62 MHz): δ = 0.95 (partly overlapping of D_2O and $OCH_2^{[18]crown-6}$, $\Delta\nu_{1/2}$ = 85 Hz), 168.29 (SO_4, $\Delta\nu_{1/2}$ = 100 Hz).

2.5 Potassium *tert*-butoxide – $K[OCMe_3]$

C_4H_9KO (112.21 g/mol): **mp** 285 °C (dec). **^{1}H NMR** (60 °C, toluene-d_8, 500.13 MHz): δ = 1.06 (s, $OCCH_3$, $^{1}J(^{1}H-^{13}C)$ = 120 Hz). **^{1}H NMR** (25 °C, THF-d_8, 300.13 MHz): δ = 0.99 (s, $OCCH_3$, $^{1}J(^{1}H-^{13}C)$ = 121 Hz). **^{13}C{^{1}H} NMR** (60 °C, toluene-d_8, 125.77 MHz): δ = 37.63 (s, $OCCH_3$), 66.62 (s, $OCCH_3$). **^{13}C{^{1}H} NMR** (25 °C, THF-d_8, 75.48 MHz): δ = 37.51 (s, $OCCH_3$), 67.22 (s, $OCCH_3$, *overlap with solvent*). **^{17}O NMR** (25 °C and also 60° C, toluene-d_8, 67.83 MHz): *(not detected)*. **IR** (ATR, 16 scans, 25 °C, cm^{-1}): $\tilde{\nu}$ = 2948 (m), 2937 (m), 2921 (m), 2896 (w), 2877 (w), 2842 (m), 1457 (w), 1446 (w), 1427 (w), 1361 (w), 1338 (m), 1224 (w), 1195 (s), 1025 (w), 973 (s), 862 (m), 736 (w). **Raman** (473 nm, 6 mW, 100 s, 50 acc., 25 °C, cm^{-1}): $\tilde{\nu}$ = 2949 (10), 2936 (7), 2920 (5), 2890 (6), 2874 (6), 2839 (7), 2710 (1), 2649 (1), 1456 (1), 1440 (4), 1395 (1), 1355 (1), 1348 (1), 1330 (1), 1325 (1), 1216 (1), 1211 (1), 1188 (7), 1005 (1), 969 (1), 856 (8), 732 (9), 474 (1), 426 (1), 347 (1), 281 (1), 156 (2), 114 (1).

2.6 1,4,7,10,13,16-Hexaoxa-*cyclo*-octadecane – [18]crown-6

$C_{12}H_{24}O_6$ (264.32 g/mol): **mp**. 41 °C. **^{1}H NMR** (25 °C, toluene-d_8, 300.13 MHz): δ = 3.49 (s, OCH_2, ^3J(^{1}H-^{1}H) = 4.6 Hz, ^1J(^{1}H-^{13}C) = 140 Hz). **^{1}H NMR** (25 °C, THF-d_8, 300.13 MHz): δ = 3.56 (s, OCH_2, ^3J(^{1}H-^{1}H) = 4.7 Hz, ^1J(^{1}H-^{13}C) = 140 Hz, *overlap with solvent signal*). **^{13}C{^{1}H} NMR** (25 °C, toluene-d_8, 75.47 MHz): δ = 71.1 (s, O*C*H_2). **^{13}C{^{1}H} NMR** (25 °C, THF-d_8, 75.47 MHz): δ = 71.6 (s, O*C*H_2). **^{17}O NMR** (25 °C, toluene-d_8, 67.83 MHz): δ = 1.83 ($\Delta\nu_{1/2}$ = 300 Hz). **Raman** (473 nm, 6 mW, 100 s, 50 acc., 25 °C, cm^{-1}): $\tilde{\nu}$ = 2984 (2), 2946 (10), 2893 (9), 2872 (8), 2840 (7), 2819 (4), 2807 (5), 2779 (1), 2764 (2), 2737 (1), 2701 (1), 2659 (1), 1490 (7), 1471 (3), 1443 (1), 1386 (1), 1372 (1), 1335 (1), 1295 (2), 1272 (2), 1257 (1), 1235 (1), 1156 (1), 1132 (3), 1107 (1), 1092 (1), 1062 (1), 1046 (1), 986 (1), 935 (1), 889 (1), 865 (2), 821 (3), 578 (1), 521 (1), 451 (1), 413 (2), 351 (1), 319 (1), 269 (1), 188 (1).

(for IR data see T. S. Cameron, A. Decken, I. Krossing, J. Passmore, J. M. Rautiainen, X. Wang, X. Zheng)[11]

2.7 Trimethylphosphinoxid – $OPMe_3$

C_3H_9OP (92.08 g/mol): **mp**. 142 °C. **^{1}H NMR** (25 °C, CD_2Cl_2, 300.13 MHz): δ = 1.36 (d, PCH_3, ^1J(^{1}H-^{13}C) = 127.2 Hz, ^2J(^{1}H-^{31}P) = 13.0 Hz). **^{13}C{^{1}H} NMR** (25 °C, CD_2Cl_2, 75.47 MHz): δ = 18.31 (d, P*C*H_3, ^2J(^{13}C-^{31}P) = 69.5 Hz). **^{17}O NMR** (25 °C, CD_2Cl_2, 67.82 MHz): δ = 64.57 (d, *O*PC, ^1J(^{17}O-^{31}P) = 150 Hz, $\Delta\nu_{1/2}$ = 35 Hz). **^{31}P{^{1}H} NMR** (25 °C, CD_2Cl_2, 121.51 MHz): δ = 36.23 (s, *P*CH). **Raman** (473 nm, 5 mW, 10 s, 10 acc., 25 °C, cm^{-1}): $\tilde{\nu}$ = 2981 (9), 2911 (10), 2802 (1), 2573 (1), 2555 (1), 1451 (1), 1432 (1), 1408 (1), 1310 (1), 1287 (1), 1145 (2), 947 (1), 934 (1), 874 (1), 859 (1), 738 (2), 664 (6), 357 (1), 316 (1), 246 (1).

(for IR data see G. Bauer, H Mikosch[12] or J. Goubeau, W. Bereger)[13]

3 Syntheses of compounds

3.1 Synthesis and characterization of Bis-1-*N*-trimethylsilyl-4-*N'*,*N'*-dimethylamino-pyridinium sulfate

Synthesis and characterization of Bis-1-*N*-trimethylsilyl-4-*N'*,*N'*-dimethylamino-pyridinium sulfate

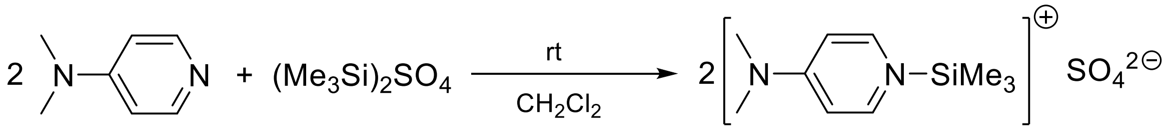

To a stirred solution of bis(trimethylsilyl)sulfate, $(Me_3SiO)_2SO_2$ (73 mg, 0.3 mmol) in 2 mL CH_2Cl_2 a solution of 4-*N*,*N*-(dimethylamino)pyridine, 4-DMAP (74 mg, 0.6 mmol) in 3 mL CH_2Cl_2 is added *via syringe*. The solution was stirred for 1 h at ambient temperature. The clear colourless solution was dried *in vacuo* for 0.5 h, leading to colourless powder.*

Several attempts to crystallize the salt failed, (e.g. cooling, heating and changing the solvent to toluene, DMSO or water.) In the case of water [DMAP-H]$_2$[SO$_4$] · 5H$_2$O (3**) could be isolated and crystallized.*

Further NMR experiments were done with 0.3 mmol $(Me_3SiO)_2SO_2$ / 0.3 mmol DMAP and 0.6 $(Me_3SiO)_2SO_2$ / 0.3mmol DMAP.

$C_{20}H_{38}N_4O_4SSi_2$ (476.78 g/mol): **mp**. 61 °C. **EA** calc. (found), %: C, 49.35 (49.14); H, 7.87 (7.42); N, 11.51 (12.20); S, 6.59 (6.21). **^{1}H NMR** (25 °C, CD_2Cl_2, 300.13 MHz): δ = 0.38 (s, SiC*H*$_3$, $^1J(^1H-^{13}C)$ = 120.5 Hz, $^2J(^1H-^{29}Si)$ = 6.7 Hz), 3.12 (s, NC*H*$_3$, ^1J(^{1}H-

^{13}C) = 138.5 Hz), 6.82 (m, *m*-C*H*), 8.13 (m, *o*-C*H*). **^{13}C{^{1}H} NMR** (25 °C, CD_2Cl_2, 75.48 MHz): *δ* = –0.02 (s, Si*C*H$_3$, ^{1}J(^{13}C-^{29}Si) = 59.3 Hz), 40.01 (s, N*C*H$_3$), 108.19 (s, *m*-*C*H), 145.75 (s, *o*-*C*H), 156.47 (s, *ipso*-*C*). **^{14}N NMR** (25 °C, CD_2Cl_2, 36.14 MHz): *δ* = –306.60 (*N*CH$_3$, Δ$v_{1/2}$ = 1100 Hz), −160.54 (*N-(*CH)$_2$, Δ$v_{1/2}$ = 450 Hz). **^{17}O NMR** (25 °C, CD_2Cl_2, 67.83 MHz): *δ* = 169.01 (*SO*$_4$, Δ$v_{1/2}$ = 210 Hz). **^{29}Si INEPT NMR** (25 °C, CD_2Cl_2, 59.52 MHz) *δ* = 24.43 (dec, *Si*CH$_3$, ^{2}J(^{29}Si-^{1}H) = 6.7 Hz). **IR** (ATR, 8 scans, 25 °C, cm^{-1}): $\tilde{v}$ = 3220 (w), 3062 (w), 2954 (w), 2902 (w), 2807 (w), 2690 (w), 2615 (w), 1635 (m), 1598 (m), 1565 (m), 1537 (w), 1521 (w), 1492 (w), 1444 (w), 1403 (m), 1376 (w), 1349 (w), 1307 (w), 1214 (s), 1184 (s), 1103 (w), 1076 (m), 1047 (m), 1025 (m), 987 (m), 943 (w), 892 (s), 838 (s), 806 (s), 779 (m), 759 (s), 690 (m), 640 (m), 617 (s), 601 (s), 584 (s). **Raman** (632 nm, 12 mW, 20 s, 10 acc, 25 °C, cm^{-1}): $\tilde{v}$ = 3087 (2), 3033 (1), 2994 (2), 2956 (3), 2899 (8), 2870 (2), 2857 (2), 2807 (2), 1640 (1), 1632 (1), 1610 (1), 1586 (2), 1564 (2), 1519 (1), 1459 (1), 1442 (1), 1414 (1), 1402 (1), 1372 (1), 1344 (1), 1304 (1), 1273 (3), 1227 (3), 1180 (1), 1073 (3), 1060 (3), 1050 (4), 1022 (1), 981 (10), 944 (5), 822 (1), 804 (1), 765 (3), 745 (8), 725 (1), 694 (1), 659 (1), 652 (1), 636 (3), 616 (4), 573 (1), 528 (1), 511 (1), 486 (1), 475 (1), 451 (1), 402 (1), 395 (1), 374 (1), 318 (1), 257 (1), 196 (1), 116 (1). **MS** (CI$^+$, m/z (%)): 243 (72), 195 (7) [M]$^+$, 165 (6) [M-(CH$_3$)$_2$]$^+$, 123 (100) [DMAP+H]$^+$.

NMR experiment with $n_{(DMAP)}$: $n_{((Me_3SiO)_2SO_2)}$

^{1}H NMR (25 °C, CD_2Cl_2, 300.13 MHz): *δ* = 0.37 (s, SiC*H*$_3$, ^{1}J(^{1}H-^{13}C) = 120.5 Hz, ^{2}J(^{1}H-^{29}Si) = 6.9 Hz), 3.21 (s, NC*H*$_3$, ^{1}J(^{1}H-^{13}C) = 139.3 Hz), 6.98 (m, *m*-C*H*), 8.13 (m, *o*-C*H*). **^{13}C{^{1}H} NMR** (25 °C, CD_2Cl_2, 75.48 MHz): *δ* = 0.03 (s, Si*C*H$_3$, ^{1}J(^{13}C-^{29}Si) = 59.8 Hz), 40.46 (s, N*C*H$_3$), 108.74 (s, *m*-*C*H), 143.59 (s, *o*-*C*H), 157.40 (s, *ipso*-*C*). **^{14}N NMR** (25 °C, CD_2Cl_2, 36.14 MHz): *δ* = –297.72 (*N*CH$_3$, Δ$v_{1/2}$ = 1750 Hz), –192.67 (*N-(*CH)$_2$, Δ$v_{1/2}$ = 45 Hz). **^{17}O NMR** (25 °C, CD_2Cl_2, 67.83 MHz): *δ* = 167.78 (*SO*$_4$, Δ$v_{1/2}$ = 185 Hz). **^{29}Si INEPT NMR** (25 °C, CD_2Cl_2, 59.52 MHz) *δ* = 27.73 (poorly resolved).

NMR experiment with 0.5$n_{(DMAP)}$: $n_{((Me_3SiO)_2SO_2)}$

^{1}H NMR (25 °C, CD_2Cl_2, 300.13 MHz): *δ* = 0.38 (s, SiC*H*$_3$, ^{1}J(^{1}H-^{13}C) = 120.6 Hz, ^{2}J(^{1}H-^{29}Si) = 7.0 Hz), 3.22 (s, NC*H*$_3$, ^{1}J(^{1}H-^{13}C) = 139.8 Hz), 6.97 (m, *m*-C*H*), 8.13 (m, *o*-C*H*).

^{13}C{^{1}H} NMR (25 °C, CD_2Cl_2, 75.48 MHz): δ = 0.08 (s, SiCH_3, $^{1}J(^{13}C\text{-}^{29}Si)$ = 60.0 Hz), 40.51 (s, NCH_3), 108.75 (s, *m*-*C*H), 143.34 (s, *o*-*C*H), 157.54 (s, *ipso*-*C*). **^{14}N NMR** (25 °C, CD_2Cl_2, 36.14 MHz): δ = –301.51 (*N*CH_3, $\Delta\nu_{1/2}$ = 1300 Hz), –200.81 (*N*-($CH)_2$, $\Delta\nu_{1/2}$ = 320 Hz). **^{17}O NMR** (25 °C, CD_2Cl_2, 67.83 MHz): δ = 164.61 (SO_4, $\Delta\nu_{1/2}$ = 140 Hz). **^{29}Si INEPT NMR** (25 °C, CD_2Cl_2, 59.52 MHz) δ = 29.72 (unresolved).

Figure S1. NMR Spectra of different DMAP/T_2SO_4 mixtures

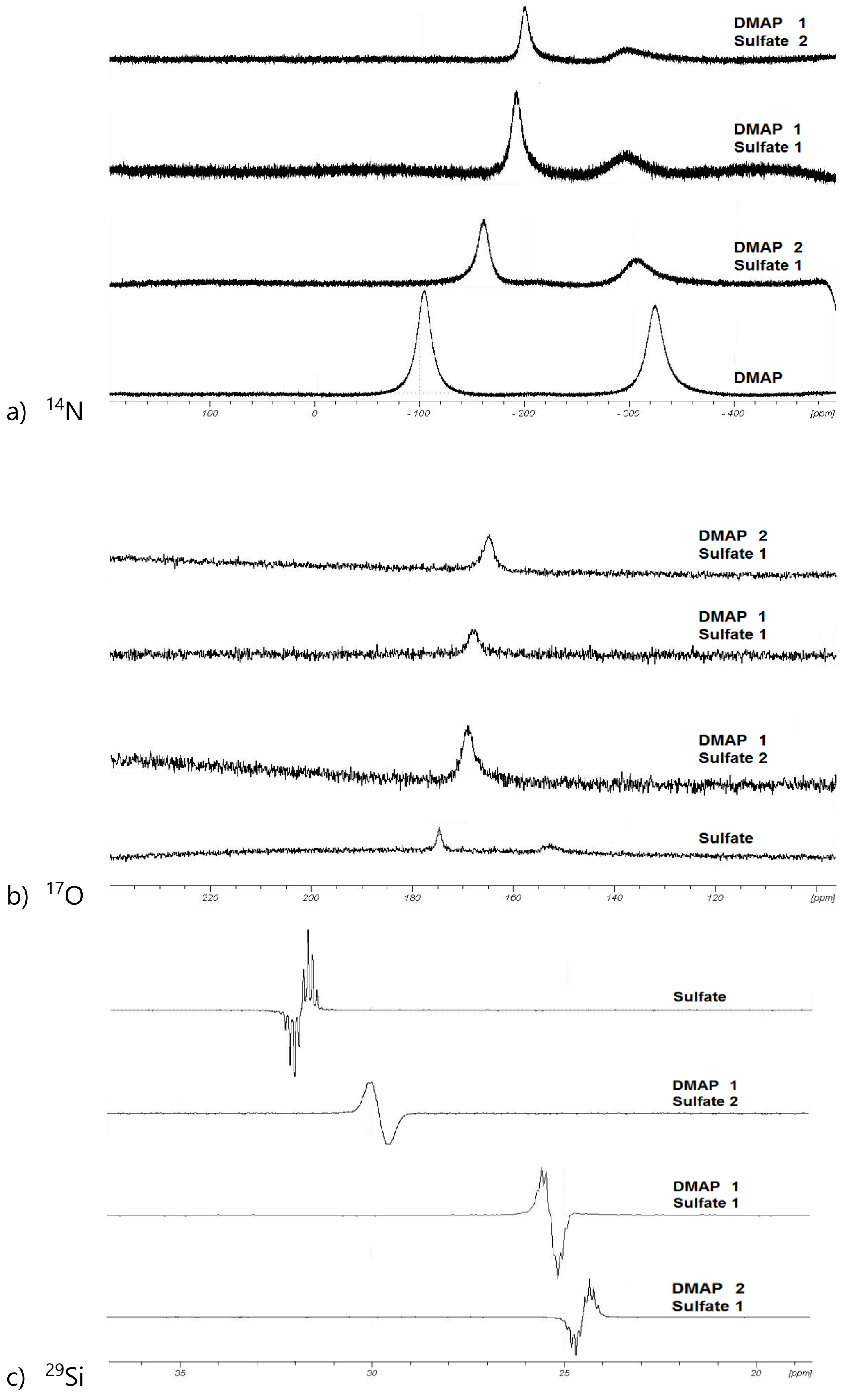

Synthesis and characterization of 4-*N′*,*N′*-dimethylamino-pyridinium sulfate $[DMAP\text{-}H]_2[SO_4]\cdot 5\ H_2O$

To a stirred solution of bis(trimethylsilyl)sulfate $(Me_3SiO)_2SO_2$ (73 mg, 0.3 mmol) in 2 mL CH_2Cl_2 a solution of 4-*N*,*N*-(dimethylamino)pyridine, DMAP (74 mg, 0.6 mmol) in 2 mL CH_2Cl_2 was added *via syringe*. The clear colourless solution was dried *in vacuo* ($1\cdot10^{-3}$ mbar) for 0.5 h at ambient temperatures, leading to ceraceous colourless substance. 2 mL of dist. H_2O was added. Single crystals suitable for X-ray structure elucidation were grown from this solution overnight yielding in 97 mg (2.2 mmol, 75 %) of colourless 4-*N*',*N*'-dimethylamino-pyridinium sulfate $[DMAP\text{-}H]_2[SO_4]\cdot 5\ H_2O$.

$C_{14}H_{22}N_4O_4S\cdot 5\ H_2O$ (432.49 g/mol): **mp**. 79 °C. **EA** calc. (found), %: C, 38.88 (38.61); H, 7.46 (7.19); N, 12.95 (12.76); S, 7.41 (7.57). **^{1}H NMR** (25 °C, D_2O, 300.13 MHz): δ = 3.14 (s, 6H, NC*H*$_3$), 6.82 (m, 2H, *m*-C*H*), 7.99 (m, 2H, *o*-C*H*), 12.55 (b, N*H*, $\Delta\nu_{1/2}$ = 84 Hz). **^{13}C{^{1}H} NMR** (25 °C, D_2O, 75.48 MHz): δ = 39.26 (s, N*C*H$_3$), 106.65 (s, *m*-*C*H), 138.23 (s, *o*-*C*H), 157.23 (s, *ipso*-*C*). **^{14}N{^{1}H} NMR** (25 °C, D_2O, 36.14 MHz): δ = –223 (*N*-(CH)$_2$, $\Delta\nu_{1/2}$ = 140 Hz), −293 (*N*CH$_3$, $\Delta\nu_{1/2}$ = 1000 Hz). **^{1}H, ^{15}N HMBC NMR** (25 °C, D_2O, 50.69 MHz) δ = –291 / 2.99 (NC*H*$_3$), −221 / 6.65 (*m*-C*H*), −221 / 7.82 (*o*-C*H*), −221 / 12.2 (N*H*, $^1J(^1H\text{-}^{15}N)$ = 95 Hz). **^{17}O NMR** (25 °C, D_2O, 67.83 MHz): δ = 166.81 ($\Delta\nu_{1/2}$ = 70 Hz). **IR** (ATR, 32 scans, 25 °C, cm^{-1}): $\tilde{\nu}$ = 3672-2196 (broad), 1687 (w), 1639 (s), 1610 (m), 1556 (s), 1446 (m), 1419 (m), 1403 (m), 1336 (w), 1280 (w), 1214 (m), 1193 (m), 1051 (s), 981 (s), 970 (s), 943 (s), 813 (s), 740 (s), 603 (s). **Raman** (473 nm, 5 mW, 5 s, 20 acc, 25 °C, cm^{-1}): $\tilde{\nu}$ = 3705-3153 (broad), 3094 (2), 2938 (7), 2873 (2), 1826 (3), 1632 (2), 1557 (5), 1517 (1), 1415 (1), 1396 (2), 1272 (1), 1215 (1), 1185 (1), 1051 (10), 989 (2), 966 (5), 938 (7), 823 (1), 740 (6), 640 (1), 600 (1), 525 (1), 508 (1), 480 (1), 449 (1), 402 (2), 278 (1), 165 (1). **MS** (CI$^+$, m/z (%)): 123 (100) [DMAP + H]$^+$.

3.2 Synthesis and characterization of trimethylsiloxytrimethylphosphonium trimethylsilylsulfate – $[Me_3SiOPMe_3][(Me_3SiO)SO_3]$

$$OPMe_3 + (Me_3SiO)_2SO_2 \xrightarrow{\text{toluene}} [Me_3SiOPMe_3][(Me_3SiO)SO_2]$$

Trimethylphosphinoxide $OPMe_3$ (36 mg, 0.4 mmol) and bis(trimethylsilyl)sulfate $(Me_3SiO)_2SO_2$ (96 mg, 0.4 mmol) were dissolved in 3 mL toluene and slightly warmed heated forming a clear and colourless solution. The reaction mixture was concentrated *in vacuo* ($1{\cdot}10^{-3}$ mbar) and crystals for X-ray structure elucidation can be obtained by cooling this solution overnight to 5 °C (water bath in refrigerator). The supernatant can be removed and the product was dried *in vacuo* ($1{\cdot}10^{-3}$ mbar) at 40 °C (oil bath) for 0.5 h yielding in colourless 103 mg (0.31 mmol, 77 %) of trimethylsiloxytrimethylphosphonium trimethylsilylsulfate.

$C_9H_{27}O_5PSSi_2$ (334.52 g/mol): **mp**. 120 °C. **EA** calc. (found), %: C, 32.31 (31.89); H, 8.14 (8.06); S, 9.59 (9.33). **^{1}H NMR** (25 °C, CD_2Cl_2, 300.13 MHz): δ = 0.05 (s, SiCH_3, $^1J(^1H-^{13}C)$ = 119 Hz, $^2J(^1H-^{29}Si)$ = 6.8 Hz), 0.33 (s, SiCH_3, $^1J(^1H-^{13}C)$ = 120 Hz, $^2J(^1H-^{29}Si)$ = 7 Hz), 1.86 (d, PCH_3, $^1J(^1H-^{13}C)$ = 130 Hz, $^2J(^1H-^{31}P)$ = 13.6 Hz). **^{1}H NMR** (25 °C, CD_2Cl_2, 500.13 MHz): δ = 0.06 (s, SiCH_3, $^1J(^1H-^{13}C)$ = 118 Hz, $^2J(^1H-^{29}Si)$ = 6.7 Hz), 0.36 (s, SiCH_3, $^1J(^1H-^{13}C)$ = 121 Hz, $^2J(^1H-^{29}Si)$ = 7 Hz), 1.66 (d, PCH_3, $^1J(^1H-^{13}C)$ = 129 Hz, $^2J(^1H-^{31}P)$ = 13.2 Hz). **^{1}H NMR** (−20 °C, CD_2Cl_2, 500.13 MHz): δ = 0.03 (s, SiCH_3, $^1J(^1H-^{13}C)$ = 118 Hz, $^2J(^1H-^{29}Si)$ = 6.6 Hz), 0.30 (s, SiCH_3, $^1J(^1H-^{13}C)$ = 120 Hz, $^2J(^1H-^{29}Si)$ = 6.6 Hz), 1.85 (d, PCH_3, $^1J(^1H-^{13}C)$ = 130 Hz, $^2J(^1H-^{31}P)$ = 13.5 Hz). **^{13}C{^{1}H} NMR** (25 °C, CD_2Cl_2, 75.47 MHz): δ = 0.63 (s, SiCH$_3$, $^1J(^{13}C-^{29}Si)$ = 60.1 Hz), 16.65 (d, PCH$_3$, $^1J(^{13}C-^{31}P)$ = 69.1 Hz). **^{29}Si INEPT NMR** (25 °C, CD_2Cl_2, 59.63 MHz): δ = 28.01 (*unresolved*). **^{31}P NMR** (25 °C, CD_2Cl_2, 121.49 MHz) δ = 63.82 (s, *P*CH$_3$). **^{17}O NMR** (25 °C, CD_2Cl_2, 67.83 MHz): 169 (br, $\Delta v_{1/2}$ = 900 Hz). **IR** (ATR, 16 scans, 25 °C, cm^{-1}): $\tilde{v}$ = 3004 (w), 2923 (w), 1421 (w), 1317 (w), 1299 (m), 1243 (m), 1139 (m), 1054 (m), 1002 (s), 958 (s), 914 (m), 867 (s), 769 (m), 671 (m), 640 (w), 617 (m), 599 (m), 586 (m), 570 (s). **Raman** (473 nm, 5 mW, 10 s, 30 acc., 25 °C, cm^{-1}): $\tilde{v}$ = 2981 (5), 2911 (10), 1419 (1), 1255 (1), 1053 (3), 857 (1), 776 (1), 702 (2), 642 (1), 619 (2), 523 (1), 376 (1), 266 (1), 246 (1), 195 (1). **MS** (CI$^+$, m/z (%)): 93 (100) $[Me_3POH]^+$, 165 (1) $[M]^+$.

3.3 Synthesis and characterization of potassium sulfate – K_2SO_4

$$2\ K[OCMe_3] + (Me_3SiO)_2SO_2 \xrightarrow{\text{DME, rt}} K_2[SO_4] + 2\ Me_3SiOCMe_3$$

Bis(trimethylsilyl)sulfate $(Me_3SiO)_2SO_2$ (48 mg, 0.2 mmol) was dissolved in 4 mL 1,2-dimethoxyethane DME. To this stirred solution a solution of potassium *tert*-butoxide $K[OCMe_3]$ (45 mg, 0.4 mmol) in DME (4 mL) was added *via syringe*. The reaction mixture gets turbid and stirred for one additional hour. The solution was filtered (G4) the colourless precipitate was washed with a small amount of *n*-hexane and dried *in vacuo* ($1\cdot10^{-3}$ mbar) at 40 °C (oilbath) for 2 h, leading to 32 mg $K_2[SO_4]$ (0.2 mmol, 92 %). The colourless filtrate was analyzed by NMR as it was.

K_2O_4S (174.26 g/mol). **ICP** calc. (found), %: S, 18.40 (18.35). **^{17}O NMR** (25 °C, D_2O, 67.83 MHz): δ = 167.09 (b, O_4S, $\Delta\nu_{1/2}$ = 90 Hz). **IR** (ATR, 32 scans, 25 °C, cm^{-1}): $\tilde{\nu}$ = 1093 (s), 981 (m), 611 (s). **Raman** (632 nm, 12 mW, 20 s, 10 acc., 25 °C, cm^{-1}): $\tilde{\nu}$ = 1144 (1), 1108 (1), 1092 (1), 983 (10), 626 (1), 619 (2), 454 (2).

$C_7H_{18}OSi$ (146.30 g/mol): **^{1}H NMR** (25 °C, CD_2Cl_2, 300.13 MHz): δ = 0.03 (s, SiCH_3, $^1J(^1H\text{-}^{13}C)$ = 117.9 Hz, $^2J(^1H\text{-}^{29}Si)$ = 6.7 Hz), 1.17 (s, CCH_3, $^1J(^1H\text{-}^{13}C)$ = 125.2 Hz), 3.25 (s, O$CH_3^{[DME]}$, $^1J(^1H\text{-}^{13}C)$ = 140.5 Hz), 3.41 (s, O$CH_2^{[DME]}$, $^1J(^1H\text{-}^{13}C)$ = 140.5 Hz). **^{13}C{^{1}H} NMR** (25 °C, CD_2Cl_2, 75.48 MHz): δ = 2.67 (s, SiCH_3), 32.27 (s, CCH_3), 55.95 (s, OCH_3), 72.35 (s, OCH_2), (CCH_3 *not observed*). **^{29}Si INEPT NMR** (25 °C, CD_2Cl_2, 59.62 MHz): δ = 6.95 (dec, *Si*CH_3, $^2J(^{29}Si\text{-}^1H)$ = 6.7 Hz).

3.4 Synthesis and NMR characterization of tris(trimethylsiloxy)oxo-sulfonium tetrakis(pentafluoro)phenylborat: $[(Me_3SiO)_3SO][B(C_6F_5)_4]$

$$[(Me_3Si)_2H][B(C_6F_5)_4] + (Me_3SiO)_2SO_2 \xrightarrow[\text{- }Me_3SiH]{\text{toluene}} [(Me_3SiO)_3SO][B(C_6F_5)_4]$$

The trimethylsilane adduct of trimethylsilylium tetrakis(pentafluorophenyl)borate $[(Me_3Si)_2H][B(C_6F_5)_4]$ (246 mg, 0.3 mmol) was suspended in 5 mL toluene. The suspension was degasified three times by a freeze-pump-thaw procedure. Bis(trimethylsilyl)sulfate $(Me_3SiO)_2SO_2$ (73 mg, 0.3 mmol) was dissolved in 5 mL toluene. This solution was added to the stirred borate solution *via* syringe. The reaction mixture was shortly heated to 60 °C (oil bath) until a typical biphasic system occurred. The clear and colourless upper toluene layer was removed *via* syringe. The viscous and colourless residue was transferred in a NMR tube with toluene-d_8.*

* *Attempts to crystallize the salt $[(Me_3SiO)_3SO)][B(C_6F_5)_4]$ failed at room temperature and also at lower temperatures 5 °C and −20 °C. Attempts to remove all the solvent in vacuo ($1\cdot10^{-3}$ mbar) at 60 °C leading to the decomposition of the salt which can be observed by the formation of a black insoluble residue. The addition of nonpolar solvents like n-hexane to precipitate the salt also failed.*

^{1}H NMR (25 °C, toluene-d_8, 300.13 MHz): δ = 0.08 (s, SiC*H*$_3$, $^1J(^1H\text{-}^{13}C)$ = 121.8 Hz, $^2J(^1H\text{-}^{29}Si)$ = 6.7 Hz). **^{11}B NMR** (25 °C, toluene-d_8, 96.29 MHz): δ = –16.16 (b, *B*$(C_6F_5)_4$, $\Delta\nu_{1/2}$ = 20 Hz). **^{13}C{^{1}H} NMR** (25 °C, toluene-d_8, 75.48 MHz): δ = –1.10 (s, SiC*H*$_3$, $^1J(^{13}C\text{-}^{29}Si)$ = 59.9 Hz), *ipso-C not observed*, 137.12 (dm, *m*-*C*F, $^1J(^{13}C\text{-}^{19}F)$ = 246 Hz), 138.79 (dm, *p*-*C*F, $^1J(^{13}C\text{-}^{19}F)$ = 246 Hz), 149.25 (dm, *o*-*C*F, $^1J(^{13}C\text{-}^{19}F)$ = 241 Hz). **^{19}F{^{1}H} NMR** (25 °C, toluene-d_8, 282.38 MHz): δ = –166.95 (t, 6F, *m*-C*F*, $^1J(^{19}F\text{-}^{13}C)$ = 245 Hz, –163.18 (t, 3F, *p*-C*F*, $^1J(^{19}F\text{-}^{13}C)$ = 246 Hz), –131.88 (b, 6F, *o*-C*F*, $^1J(^{19}F\text{-}^{13}C)$ = 242 Hz). **^{17}O NMR** (25 °C, toluene-d_8, 67.83 MHz): δ = 157.99 (b, *O*SiMe$_3$, $\Delta\nu_{1/2}$ = 900 Hz). **^{29}Si INEPT NMR** (25 °C, toluene-d_8, 59.52 MHz) δ = 55.10 (dec, *Si*CH$_3$, $^2J(^{29}Si\text{-}^1H)$ = 6.7 Hz).

$$[(Me_3Si)_2H][B(C_6F_5)_4] + (Me_3SiO)_2SO_2 \xrightarrow[\text{- } Me_3SiH]{\text{1,2-DCB}} [(Me_3SiO)_3SO][B(C_6F_5)_4]$$

The trimethylsilane adduct of trimethylsilylium tetrakis(pentafluorophenyl)borate $[(Me_3Si)_2H][B(C_6F_5)_4]$ (83 mg, 0.1 mmol) and bis(trimethylsilyl)sulfate (73 mg, 0.3 mmol) were suspended* in 0.5 mL 1,2-DCB in a Young valve NMR tube. The deuterated solvent toluene-d_8 was added in a second smaller inner precision glass tube.

** Forming a more homogenous solution by warming of the 1,2-DCB suspension to 60 °C leading to the decomposition of the mixture which can be observed by the darkening of the reaction mixture.*

^{1}H NMR (25 °C, 1,2-DCB, ref. ext. toluene-d_8, 300.13 MHz): δ = −0.06 (s) −0.03 (b, $\Delta\nu_{1/2}$ = 7 Hz), 0.18 (s, $(SiCH_3)_2$, $^1J(^1H\text{-}^{13}C)$ = 119.8 Hz), 6.73 (m, o-C$H^{[1,2\text{-DCB}]}$), 7.04 (m, o-C$H^{[1,2\text{-DCB}]}$). **^{11}B NMR** (25 °C, 1,2-DCB, 96.29 MHz): δ = –16.14 (b, $B(C_6F_5)_4$, $\Delta\nu_{1/2}$ = 22 Hz). **^{13}C{^{1}H} NMR** (25 °C, 1,2-DCB, ref. ext. toluene-[D_8], 75.48 MHz): δ = –2.72 (b, $\Delta\nu_{1/2}$ = 6 Hz), –0.71 (s, $(SiCH_3)_2$, $^1J(^{13}C\text{-}^{29}Si)$ = 60.4 Hz), –0.01 (s), 118.28 (b, *ipso*-C^F), 127.77 (s, m-$CH^{[1,2\text{-DCB}]}$), 130.54 (s, o-$CH^{[1,2\text{-DCB}]}$), 132.62 (s, *ipso*-$C^{Cl[1,2\text{-DCB}]}$), 136.54 (dm, m-CF, $^1J(^{13}C\text{-}^{19}F)$ = 246 Hz), 138.65 (dm, p-CF, $^1J(^{13}C\text{-}^{19}F)$ = 245 Hz), 148.93 (dm, o-CF, $^1J(^{13}C\text{-}^{19}F)$ = 241 Hz). **^{17}O NMR** (25 °C, 1,2-DCB, 67.83 MHz): δ = 158.52 (b, $OSiMe_3$). **^{19}F{^{1}H} NMR** (25 °C, 1,2-DCB, 282.38 MHz): δ = –166.45 (t, 6F, m-CF, $^1J(^{19}F\text{-}^{13}C)$ = 247 Hz, −162.67 (t, 3F, p-CF, $^1J(^{19}F\text{-}^{13}C)$ = 246 Hz), –131.70 (b, 6F, o-CF, $^1J(^{19}F\text{-}^{13}C)$ = 243 Hz). **^{29}Si INEPT NMR** (25 °C, 1,2-DCB, 59.52 MHz) δ = 48.28 (dec, $SiCH_3$, $^2J(^{29}Si\text{-}^1H)$ = 6.9 Hz).

3.5 Synthesis and NMR characterization of tris(trimethylsiloxy)oxo-sulfonium hexabromo pentahydro undecaborat: $[(Me_3SiO)_3SO][CHB_{11}Br_6H_5]$

Trimethylsilyl hexachloro pentahydro undecaborat $[Me_3Si][CHB_{11}Br_6H_5]$ was prepared by a literature known synthesis.[6–10]

209 mg (0.49 mmol) $[Me_3Si][CHB_{11}Br_6H_5]$ was suspended in 2 mL toluene. 76 mg (0.31 mmol) bis(trimethylsilyl)sulfate $[Me_3SiO]_2SO_2$ was dissolved in 1 mL toluene and added via syringe to the suspended $[Me_3Si][CHB_{11}Br_6H_5]$. Within a few minutes the borate dissolved and it formed a two phase system with clear upper phase and highly viscous orange oil. The two phase system was heated in a water bath to 60 °C and treated for 30 min with ultrasonic. The formed homogenies orange solution was cooled overnight from 60 °C to 25 °C. Colourless crystals suitable for X-ray structure elucidation were obtained. The supernatant is removed by syringe and discarded. The remaining crystals are dried ($1{\cdot}10^{-3}$ mbar) at 40 °C for 30 min, yielding 141 mg (0.21 mmol, 68 %) of tris(trimethylsiloxy)sulfinylium hexabromo pentahydro undecaborat $[(Me_3SiO)_3SO][CHB_{11}Br_6H_5]$.

$C_{10}H_{33}B_{11}Br_6Si_3SO_4$ (944.05 g·mol^{-1}): **mp**. 114 °C, 160 °C (dec.). **EA** calc. (found), %: C, 12.89 (13.36); H, 3.57 (3.36); S, 3.44 (3.18). **^{1}H NMR** (25 °C, CD_2Cl_2, 500.13 MHz): δ = 0.40 (s, 27H, C*H*$_3$, $^1J(^1H\text{-}^{13}C)$ = 121.9 Hz, $^2J(^1H\text{-}^{29}Si)$ = 7.6 Hz), 2.18 - 3.84 (br, 6H, C*H*$B_{11}Br_6$*H*$_5$). **^{11}B NMR** (25 °C, C_6D_6, 160.5 MHz): δ = –0.9 (br, 1B, *B*Cl, $\Delta\nu_{1/2}$ = 120 Hz), –9.1 (s, 5B, *B*Cl, $\Delta\nu_{1/2}$ = 72 Hz), –19.8 (d, 5B, *B*H, $^2J(^1H\text{-}^{11}B)$ = 153 Hz). **^{13}C{^{1}H} NMR** (25 °C, C_6D_6, 125.8 MHz): δ = 0.6 (s, Si*C*H$_3$), 42.2 (s, $CHB_{11}Br_6H_5$). **^{29}Si INEPT NMR** (25 °C, C_6D_6, 99.36 MHz): δ = 54.1 (dec, *Si*CH$_3$, $^2J(^{29}Si\text{-}^1H)$ = 7.6 Hz). **Raman** (785 nm, 633 nm, 532 nm, 473 nm, 25 °C, cm^{-1}): fluorescence. **IR** (ATR, 32 scans, 25 °C, cm^{-1}): 466 (w), 484 (w), 517 (m), 606 (m), 622 (m), 635 (m), 676 (m), 717 (m), 746 (m), 767 (s), 808 (vs), 857 (s), 917 (w), 933 (m), 956 (m), 991 (m), 1004 (m), 1065 (s), 1133 (w), 1261 (m), 1339 (m), 1391 (w), 1420 (w), 1467 (w), 2594 (m), 2905 (w), 2971 (w), 3049 (w).

4 Structure elucidation

X-ray Structure Determination: X-ray quality crystals were selected in Fomblin YR-1800 perfluoroether (Alfa Aesar) at ambient temperature. The samples were cooled to 123(2) K during measurement. The data were collected on a Bruker D8 Quest diffractometer or a Bruker Kappa Apex II diffractometer using Mo K_α radiation (λ = 0.71073 Å). The structures were solved by iterative methods (SHELXT)[14] and refined by full matrix least squares procedures (SHELXL).[15] Semi-empirical absorption corrections were applied (SADABS).[16] All non-hydrogen atoms were refined anisotropically, hydrogen atoms were included in the refinement at calculated positions using a riding model.

Table S2: Crystallographic details.

**Compound**	T_2SO_4
Chem. Formula	$C_6H_{18}O_4SSi_2$
Formula weight [g/mol]	242.44
Colour	colourless
Crystal system	monoclinic
Space group	*C*2/*c*
a [Å]	21.003(2)
b [Å]	20.985(2)
c [Å]	12.773(2)
α [°]	90
β [°]	112.884(3)
γ [°]	90
V [Å^3]	5186.9(9)
Z	16
$\rho_{calcd.}$ [g/cm^3]	1.242
μ [mm^{-1}]	0.42
$\lambda_{MoK\alpha}$ [Å]	0.71073
T [K]	123
Measured reflections	44838
Independent reflections	9347
Reflections with $I > 2\sigma(I)$	6395
R_{int}	0.053
F(000)	2080
$R_1(R[F^2>2\sigma(F^2)])$	0.060
$wR_2(F^2)$	0.123
GooF	1.078
No. of Parameters	275
CCDC #	2025532

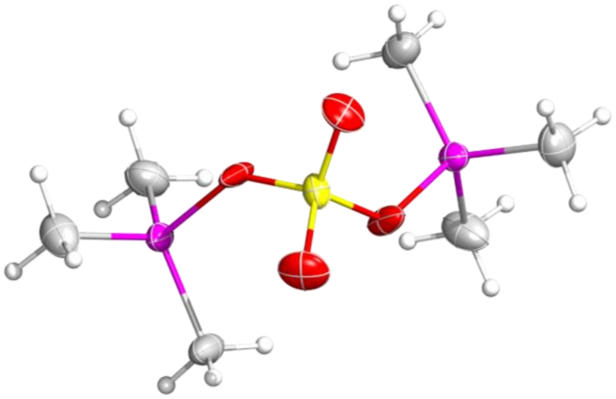

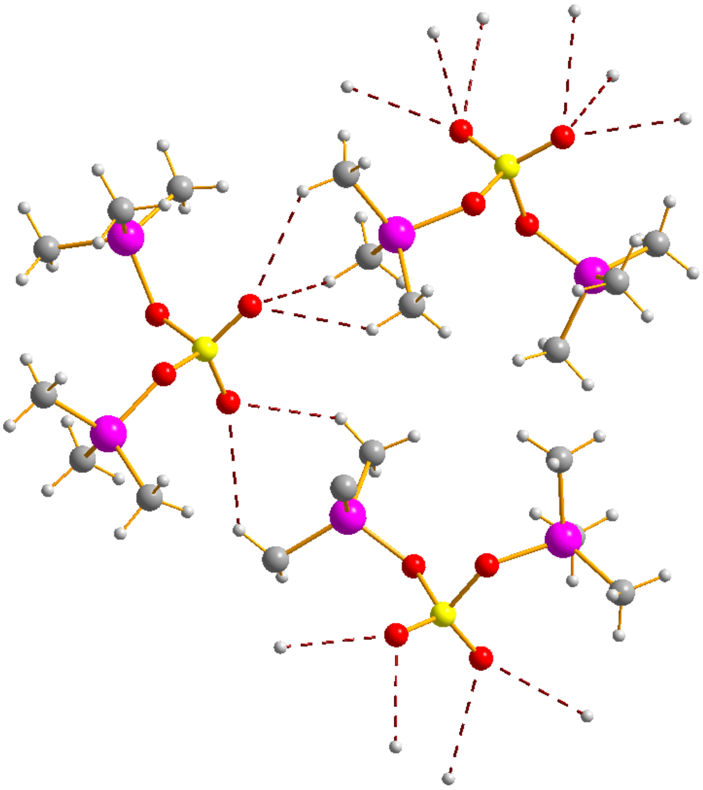

Figure S2. Weak H···O- van der Waals interactions in T_2SO_4. Color code: yellow S, violet Si, grey C, white H, and red O.

Compound	$[Me_3PO\text{-}T][TSO_4]$
Chem. Formula	$C_9H_{27}O_5PSSi_2$
Formula weight [g/mol]	334.51
Colour	colourless
Crystal system	orthorhombic
Space group	*Pbca*
a [Å]	13.133(1)
b [Å]	18.569(2)
c [Å]	29.798(2)
α [°]	90
β [°]	90
γ [°]	90
V [Å^3]	7266.2(8)
Z	16
$\rho_{calcd.}$ [g/cm^3]	1.223
μ [mm^{-1}]	0.41
$\lambda_{MoK\alpha}$ [Å]	0.71073
T [K]	123
Measured reflections	110692
Independent reflections	12572
Reflections with $I > 2\sigma(I)$	8551
R_{int}	0.069
F(000)	2880
$R_1(R[F^2>2\sigma(F^2)])$	0.050
$wR_2(F^2)$	0.124
GooF	1.074
No. of Parameters	522
CCDC #	2025534

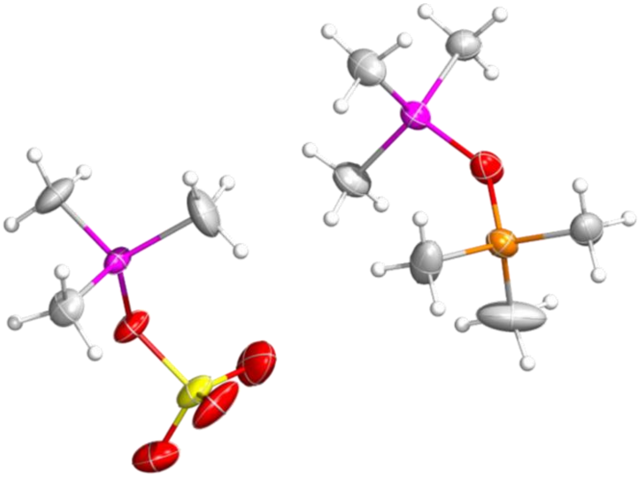

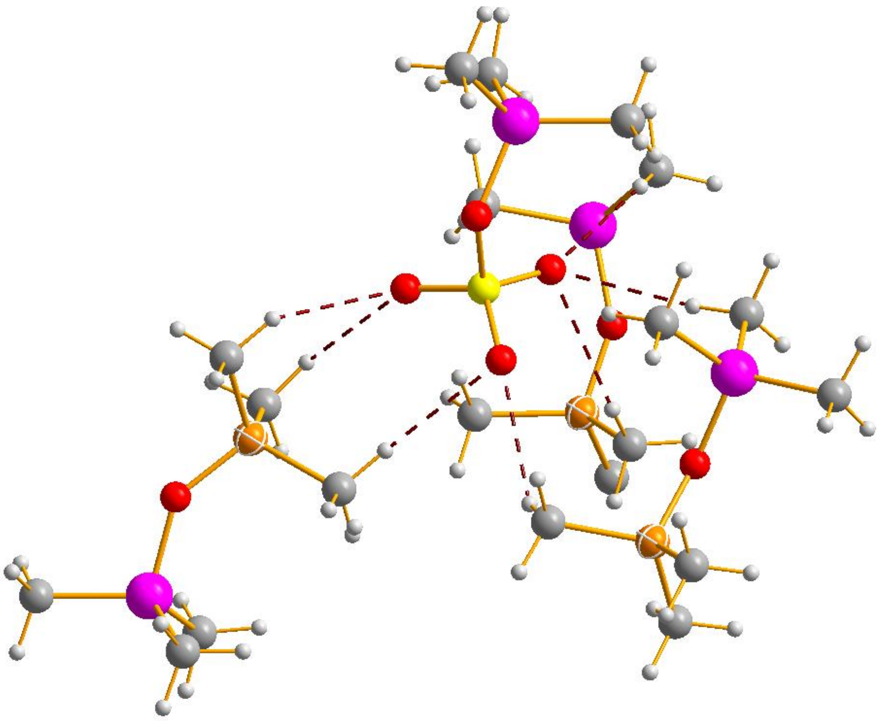

Figure S3. Weak H···O- van der Waals interactions in [Me_3PO-T][TSO_4]. Color code: yellow S, violet Si, grey C, white H, and red O.

Compound	**$[T_3SO_4][CHB_{11}Br_6H_6]$**
Chem. Formula	$C_{10}H_{33}B_{11}Br_6O_4SSi_3$
Formula weight [g/mol]	944.05
Colour	colourless
Crystal system	orthorombic
Space group	$P2_12_12_1$
a [Å]	10.1609(8)
b [Å]	14.688(2)
c [Å]	22.478(2)
α [°]	90
β [°]	90
γ [°]	90
V [Å^3]	3354.6(4)
Z	4
$\rho_{calcd.}$ [g/cm^3]	1.846
μ [mm^{-1}]	7.37
$\lambda_{MoK\alpha}$ [Å]	0.71073
T [K]	123
Measured reflections	4501
Independent reflections	4501
Reflections with $I > 2\sigma(I)$	3542
R_{int}	0.144
F(000)	1792
$R_1(R[F^2>2\sigma(F^2)])$	0.084
$wR_2(F^2)$	0.129
GooF	1.08
No. of Parameters	326
CCDC #	2025531

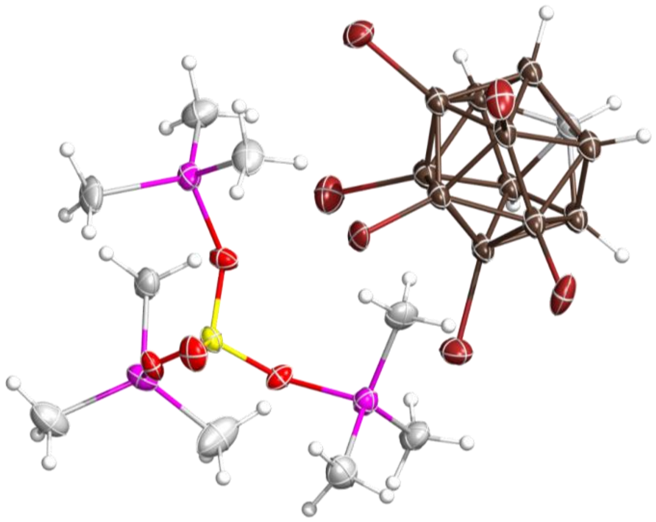

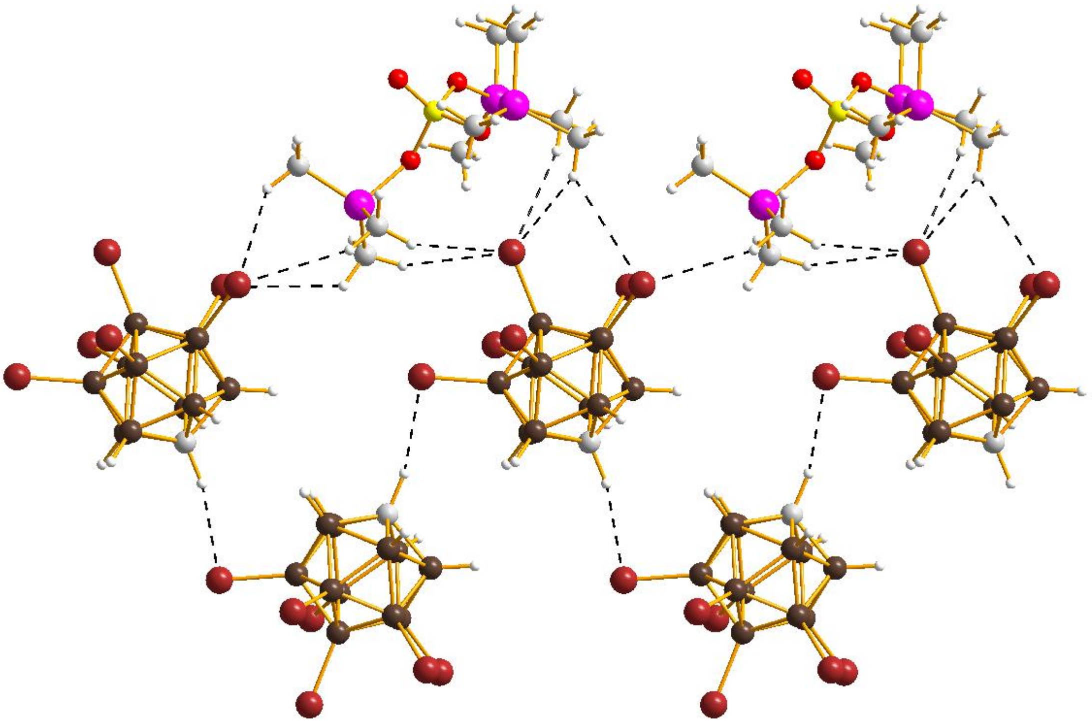

Figure S4. Weak H···Br- and H···O- van der Waals interactions in $[T_3SO_4][CHB_{11}Br_6H_6]$. Color code: yellow S, violet Si, grey C, white H, brown B, red brown Br, and red O.

Compound	TOP_2-S_2O_7
Chem. Formula	$C_{12}H_{36}O_9P_2S_2Si_2$
Formula weight [g/mol]	506.65
Colour	colourless
Crystal system	monoclinic
Space group	*Pc*
a [Å]	11.658(2)
b [Å]	8.146(1)
c [Å]	13.720(2)
α [°]	90
β [°]	104.544(5)
γ [°]	90
V [Å^3]	1261.0(2)
Z	2
$\rho_{calcd.}$ [g/cm^3]	1.334
μ [mm^{-1}]	0.47
$\lambda_{MoK\alpha}$ [Å]	0.71073
T [K]	123
Measured reflections	20834
Independent reflections	7551
Reflections with $I > 2\sigma(I)$	5656
R_{int}	0.037
F(000)	540
$R_1(R[F^2>2\sigma(F^2)])$	0.044
$wR_2(F^2)$	0.108
GooF	1.020
No. of Parameters	257
CCDC #	2025533

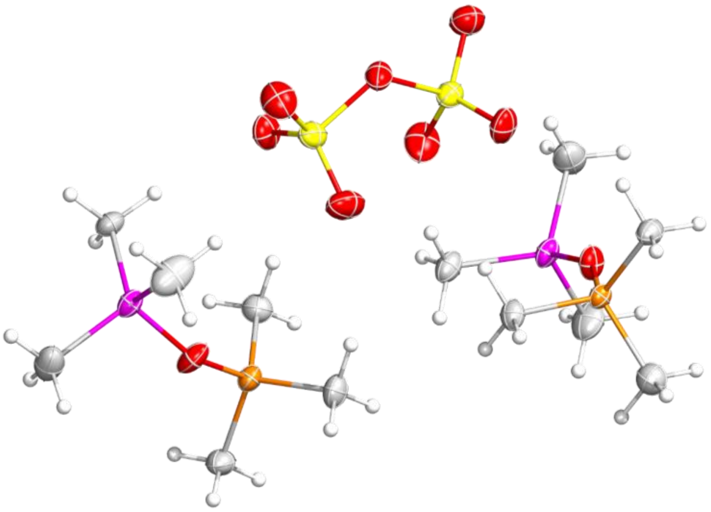

**Compound**	**(Cs·[18]crown-6)$_2$-S_2O_7**
Chem. Formula	$C_{24}H_{28}Cs_2O_{19}S_2$
Formula weight [g/mol]	970.56
Colour	colourless
Crystal system	monoclinic
Space group	$P2_1/n$
a [Å]	10.588(1)
b [Å]	31.331(2)
c [Å]	11.168(1)
α [°]	90
β [°]	98.559(2)
γ [°]	90
V [Å^3]	3662.9(3)
Z	4
$\rho_{calcd.}$ [g/cm^3]	1.760
μ [mm^{-1}]	2.18
$\lambda_{MoK\alpha}$ [Å]	0.71073
T [K]	123
Measured reflections	150168
Independent reflections	13222
Reflections with $I > 2\sigma(I)$	11593
R_{int}	0.047
F(000)	1944
$R_1(R[F^2>2\sigma(F^2)])$	0.034
w$R_2(F^2)$	0.069
GooF	1.163
No. of Parameters	587
CCDC #	2025530

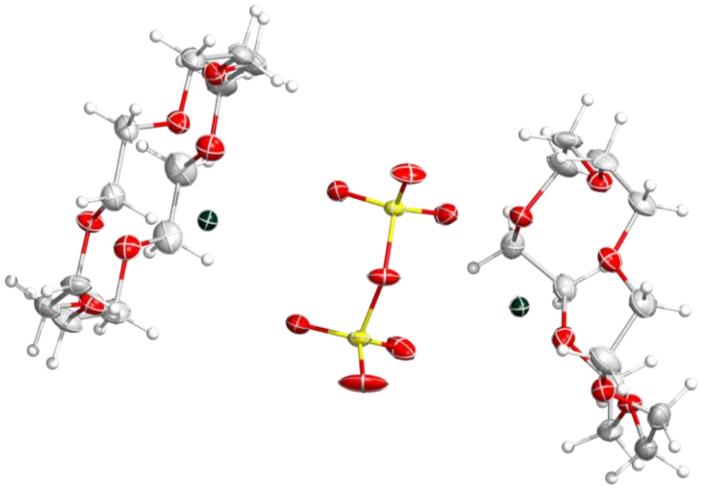

5 Comparison of experimental spectroscopic data

5.1 Comparison of NMR data

The following table contains experimental NMR data.

Table S3: Experimental NMR data.

	Chemical shift [ppm]		Coupling constant [Hz]	
	**^{1}H**	**^{29}Si**	**$^{1}J(^{13}C,^{1}H)$**	**$^{2}J(^{29}Si,^{1}H)$**
**$[TSO_4]^-$**	0.05	28.0	119.0	6.8
**T_2SO_4**	0.18	31.9	120.7	7.1
**$[T_3SO_4]^+$**	0.40	54.1	121.9	7.6

T = Me_3Si

5.2 Comparison of structural data

Table S4: Experimental structural data (in Å).

	**Si−O**	**(Si)O−S**	**O−S**
**$[TSO_4]^-$**	1.684(4)	1.587(3)	1.442(4)
**T_2SO_4**	1.726(8)	1.51(1)	1.44(2)
**$[T_3SO_4]^+$**	1.771(9)	1.492(8)	1.410(9)

T = Me_3Si

6 Computational Details

All computations were carried out using Gaussian09[17] as well as the standalone version of NBO 6.0.[18–21]

Methods. DFT calculations were carried out using the hybrid DFT functional PBE0[22–25] and an aug-cc-pwCVDZ[26–28] basis set (notation PBE0/aug-cc-pwCVDZ). All structures were fully optimized and confirmed as minima by frequency analyses. Only lowest lying isomers are discussed. A natural bond orbital analysis (NBO) was performed at the same level as mentioned above, to study the charge distribution, bond polarization and hybridization effects.[29–32] Chemical shifts and coupling constants were derived by the GIAO method.[33–37] The calculated absolute shifts ($\sigma_{calc,X}$) were referenced to the calculated absolute shifts of $BF_3{\cdot}Et_2O$ (^{11}B, σ_{ref} = 104.6548 ppm), Me_4Si (^{1}H, σ_{ref} = 30.96 ppm; ^{13}C, σ_{ref} = 187.90 ppm, ^{29}Si, σ_{ref} = 391.81 ppm) or H_2O (^{17}O, σ_{ref} = 333.91 ppm)

Please note that all computations were carried out for single, isolated molecules in the gas phase (ideal gas approximation). There may well be significant differences between gas phase and condensed phase.

$OPMe_3$ – C_{3V}

E_0 = −536.0365335

$[Me_3SiOPMe_3]^+$ – C_1

E_0 = −944.8788039

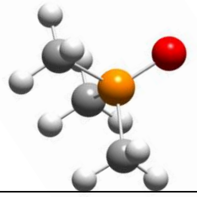

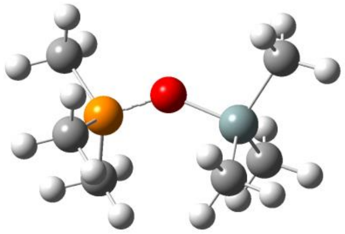

P	0.000000	0.000000	0.179924		P	−1.505407	−0.000427	−0.076835
O	0.000000	0.000000	1.679308		O	−0.041157	0.004893	−0.628076
C	0.000000	1.657643	−0.552771		C	−1.941613	1.604736	0.582528
H	0.889077	2.198241	−0.205887		H	−1.807871	2.367656	−0.193693
H	0.000000	1.619647	−1.649373		H	−2.990509	1.597541	0.905260
H	−0.889077	2.198241	−0.205887		H	−1.303189	1.844821	1.440763
C	−1.435561	−0.828821	−0.552771		C	−1.723461	−1.231193	1.205021
H	−1.402656	−0.809824	−1.649373		H	−2.776432	−1.252950	1.513323
H	−1.459194	−1.869084	−0.205887		H	−1.439358	−2.218906	0.822989
H	−2.348271	−0.329157	−0.205887		H	−1.104604	−0.985132	2.076162
C	1.435561	−0.828821	−0.552771		C	−2.576750	−0.384295	−1.452450
H	1.402656	−0.809824	−1.649373		H	−2.442055	0.367399	−2.239374
H	2.348271	−0.329157	−0.205887		H	−2.321104	−1.373207	−1.851200
H	1.459194	−1.869084	−0.205887		H	−3.623372	−0.382945	−1.123344
					Si	1.623180	0.002318	−0.038756
					C	1.964200	−1.729952	0.536240

					H	3.022559	−1.817198	0.821356
					H	1.371789	−2.012691	1.416437
					H	1.780357	−2.462894	−0.260371
					C	2.586767	0.488329	−1.539901
					H	3.660885	0.504415	−1.307667
					H	2.435154	−0.225332	−2.360124
					H	2.308178	1.488639	−1.895741
					C	1.713520	1.246425	1.341374
					H	2.754581	1.319672	1.687647
					H	1.412578	2.250590	1.014616
					H	1.112267	0.961027	2.215794

DMAP – C_s

E_0 = −381.8595551

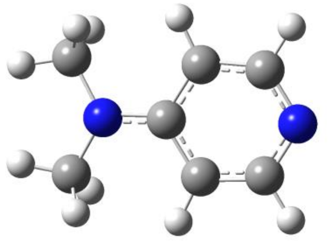

[DMAP-SiMe$_3$]$^+$ - C_1

E_0 = −790.7091344

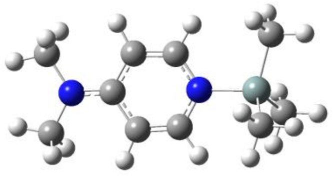

C	−0.025412	-1.950655	1.128641		C	−1.132829	0.026757	0.000000
C	0.015991	−0.565466	1.194598		C	−1.126311	−1.339827	0.000000
C	0.049379	0.182659	0.000000		C	0.108793	−2.048240	0.000000
C	0.015991	−0.565466	−1.194598		C	1.282592	−1.244424	0.000000
C	−0.025412	−1.950655	−1.128641		C	1.184332	0.120180	0.000000
H	−0.048161	−2.524019	2.058795		H	−2.075132	0.571762	0.000000
H	0.019694	−0.085064	2.169611		H	−2.079551	−1.858757	0.000000
H	0.019694	−0.085064	−2.169611		H	2.273967	−1.685994	0.000000
H	−0.048161	−2.524019	−2.058795		H	2.078077	0.738793	0.000000
N	0.117107	1.551161	0.000000		N	0.161240	−3.385318	0.000000
N	−0.044427	−2.665256	0.000000		N	−0.001685	0.777122	0.000000
C	−0.025412	2.271532	1.243818		C	−1.065137	−4.169871	0.000000
H	0.101861	3.341006	1.053137		H	−0.806661	−5.230144	0.000000
H	−1.013329	2.118178	1.711231		H	−1.667070	−3.964578	0.895636
H	0.746416	1.968789	1.964827		H	−1.667070	−3.964578	−0.895636
C	−0.025412	2.271532	−1.243818		C	1.445896	−4.069878	0.000000
H	0.746416	1.968789	−1.964827		H	2.029883	−3.817169	0.895479
H	−1.013329	2.118178	−1.711231		H	1.272604	−5.147378	0.000000
H	0.101861	3.341006	−1.053137		H	2.029883	−3.817169	−0.895479
C	−0.025412	−1.950655	1.128641		Si	−0.135555	2.641405	0.000000
C	0.015991	−0.565466	1.194598		C	−1.065137	3.062963	−1.552697
C	0.049379	0.182659	0.000000		H	−2.075068	2.632127	−1.569869
C	0.015991	−0.565466	−1.194598		H	−1.180008	4.153884	−1.624719
					H	−0.531401	2.725384	−2.450705
					C	1.608860	3.279508	0.000000
					H	1.562501	4.378018	0.000000

					H	2.173433	2.985660	0.894743
					H	2.173433	2.985660	−0.894743
					C	−1.065137	3.062963	1.552697
					H	−0.531401	2.725384	2.450705
					H	−1.180008	4.153884	1.624719
					H	−2.075068	2.632127	1.569869

$[Me_3SiOSO_3]^-$ – C_1

$E_0 = -1107.8285514$

$(Me_3SiO)_2SO_2$ – C_2

$E_0 = -1516.8044652$

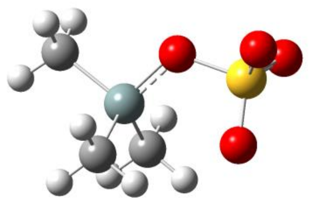

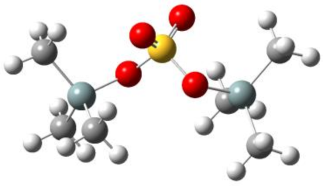

S	−1.580411	−0.006164	−0.049728	S	0.000000	0.000000	1.075359
O	−2.342666	−1.088016	−0.668205	O	−0.406052	1.184668	1.790367
O	−1.163562	−0.261032	1.344389	O	0.406052	−1.184668	1.790367
O	−2.101858	1.340160	−0.285573	O	1.163589	0.367792	0.078228
O	−0.130244	−0.026449	−0.882865	O	−1.163589	−0.367792	0.078228
Si	1.323004	−0.000776	−0.040146	Si	−1.543143	−1.992553	−0.440102
C	1.658508	−1.595840	0.884411	Si	1.543143	1.992553	−0.440102
H	2.666070	−1.589967	1.327659	C	2.290284	2.910799	0.992191
H	0.913447	−1.726132	1.678276	H	2.625787	3.907402	0.671354
H	1.588455	−2.455861	0.203453	H	1.558932	3.034183	1.800005
C	1.486953	1.482659	1.090366	H	3.160843	2.373836	1.391631
H	2.505178	1.555090	1.501954	C	−2.290284	−2.910799	0.992191
H	1.268964	2.407645	0.538795	H	−2.625787	−3.907402	0.671354
H	0.766573	1.401776	1.913215	H	−1.558932	−3.034183	1.800005
C	2.589534	0.153751	−1.426651	H	−3.160843	−2.373836	1.391631
H	3.616882	0.162020	−1.032437	C	0.000000	−2.795681	−1.098614
H	2.501322	−0.687283	−2.128820	H	−0.240442	−3.783705	−1.516283
H	2.434306	1.081497	−1.995143	H	0.451872	−2.192387	−1.896846

					H	0.742974	−2.930181	−0.303290
					C	2.787450	1.654794	−1.783204
					H	3.169717	2.598678	−2.196530
					H	3.642990	1.086234	−1.395545
					H	2.340895	1.082538	−2.606942
					C	−2.787450	−1.654794	−1.783204
					H	−3.169717	−2.598678	−2.196530
					H	−3.642990	−1.086234	−1.395545
					H	−2.340895	−1.082538	−2.606942
					C	0.000000	2.795681	−1.098614
					H	0.240442	3.783705	−1.516283
					H	−0.451872	2.192387	−1.896846
					H	−0.742974	2.930181	−0.303290

[(Me$_3$SiO)$_3$SO]$^+$ - C$_1$

E_0 = −1925.6186775

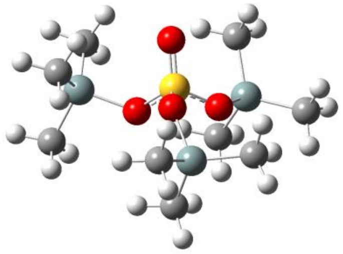

S	−0.031946	0.013587	−0.713187
O	1.014488	−0.933403	−0.137988
O	−0.589704	−0.382309	−1.966949
O	0.681704	1.347987	−0.819534
O	−1.073770	0.150491	0.399918
Si	−2.819645	0.609473	0.185570
Si	1.975668	2.137688	0.171400
C	3.551474	1.306628	−0.329950
H	4.399386	1.810586	0.155760
H	3.574271	0.251577	−0.031909
H	3.706275	1.368863	−1.414778
C	−3.647079	−0.893597	−0.508410
H	−4.724766	−0.700869	−0.609312
H	−3.259417	−1.143823	−1.503576
H	−3.530410	−1.762676	0.152116
C	−2.835151	2.079453	−0.940307
H	−3.865107	2.455536	−1.023053
H	−2.217309	2.899209	−0.551765
H	−2.494168	1.826541	−1.951965

C	1.816557	3.880824	−0.421326
H	2.585644	4.504525	0.056130
H	1.961418	3.946252	−1.507263
H	0.837387	4.309740	−0.173635
C	−3.264821	0.981074	1.942438
H	−4.331716	1.238559	2.004144
H	−3.095688	0.114186	2.593931
H	−2.694190	1.831452	2.336674
C	1.508339	1.888745	1.948564
H	2.229803	2.424178	2.582452
H	0.512867	2.293868	2.169844
H	1.531530	0.832717	2.245140
Si	0.925921	−2.717140	0.199188
C	0.153680	−2.828582	1.879420
H	0.124949	−3.879541	2.200669
H	0.727439	−2.268642	2.628863
H	−0.878132	−2.455162	1.878852
C	2.725164	−3.140358	0.151976
H	2.858032	−4.209235	0.371004
H	3.156458	−2.947799	−0.838660
H	3.295352	−2.576277	0.901195
C	−0.081585	−3.484293	−1.149523
H	−1.137180	−3.188665	−1.108128
H	0.309277	−3.236804	−2.144060
H	−0.039295	−4.577471	−1.034757

$[SO_3]^{2-}$ – C_{3V}

E_0 = −623.4333784

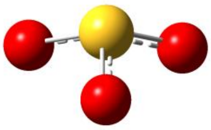

$[SO_4]^{2-}$ – T_d

E_0 = −698.6412194

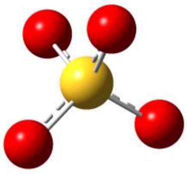

S	0.000000	0.000000	0.337868		S	0.000000	0.000000	0.000000
O	0.000000	1.448145	−0.225245		O	0.874485	0.874485	0.874485
O	1.254130	−0.724073	−0.225245		O	−0.874485	−0.874485	0.874485
O	−1.254130	−0.724073	−0.225245		O	−0.874485	0.874485	−0.874485
					O	0.874485	−0.874485	−0.874485

$[(Me_3SiO)SO_2]^-$ – C_1

E_0 = −1032.6485137

$[(Me_3Si)SO_3]^-$ – C_1

E_0 = −1032.6193021

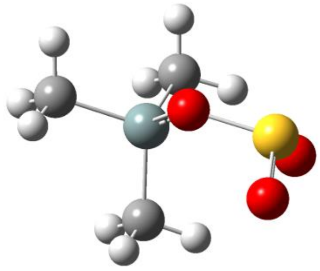

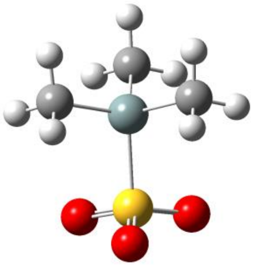

S	−1.840350	0.056856	−0.457895	S	−1.209951	0.000507	0.001030
O	−1.494776	1.238227	0.405061	O	−1.594489	−0.538741	−1.329839
O	−2.432368	−1.061672	0.326872	O	−1.595761	1.422334	0.198829
O	−0.217112	−0.578862	−0.837769	O	−1.596648	−0.882278	1.132716
Si	1.119871	−0.050860	0.002166	Si	0.987666	−0.000349	0.000029
C	1.575851	1.733956	−0.380361	C	1.584090	−1.767887	−0.241179
H	2.514483	2.024310	0.116552	H	1.195713	−2.164953	−1.188641
H	1.704998	1.876612	−1.463314	H	2.682783	−1.835973	−0.253982
H	0.767835	2.394204	−0.041552	H	1.201305	−2.403304	0.568714
C	2.530034	−1.140540	−0.628865	C	1.591148	0.675121	1.648908
H	3.489202	−0.870137	−0.160501	H	2.690194	0.698532	1.708105
H	2.329866	−2.199319	−0.410815	H	1.209196	1.694310	1.795963
H	2.643098	−1.040192	−1.718094	H	1.207988	0.053513	2.469301
C	0.994160	−0.285744	1.860404	C	1.584554	1.091158	−1.410911
H	0.733553	−1.326826	2.097375	H	1.205019	2.112931	−1.276169
H	1.942645	−0.039837	2.363159	H	2.683232	1.127883	−1.469975
H	0.195506	0.355932	2.252813	H	1.192896	0.712973	−2.364760

$[(Me_3SiO)_2SO]$ – C_1

E_0 = −1441.6382028

$[(Me_3SiO)S(O_2)SiMe_3]$ – C_1

E_0 = −1441.6071661

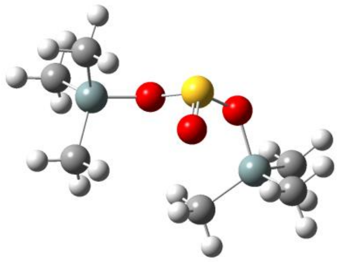

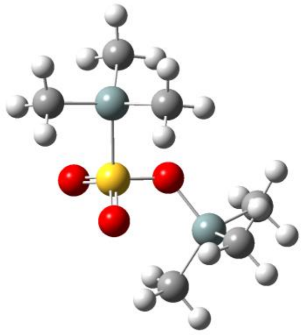

S	0.014667	−1.131957	−0.660302	S	−0.429602	−0.853180	0.002195
O	−0.068859	−1.561527	0.747775	O	−0.049967	−1.161907	1.382829
O	0.946229	0.217561	−0.685592	O	0.685180	0.141230	−0.603454
O	−1.367442	−0.407819	−1.055767	O	−0.595397	−1.959210	−0.931534
Si	2.509248	0.281037	0.041233	Si	−2.249837	0.441099	−0.034663
Si	−2.503874	0.302877	0.051166	C	−3.574276	−0.681718	0.640105
C	3.385677	−1.340184	−0.255905	H	−3.331466	−1.008037	1.659366
H	4.414282	−1.289356	0.128634	H	−4.539215	−0.155725	0.668823
H	2.879688	−2.169724	0.255567	H	−3.688139	−1.573054	0.010107
H	3.440646	−1.576515	−1.327142	C	−2.495013	0.900701	−1.822938
C	2.323668	0.632837	1.860627	H	−3.408410	1.500633	−1.943306
H	3.308226	0.727540	2.340296	H	−1.646628	1.487189	−2.197421
H	1.774971	1.569104	2.028825	H	−2.590313	−0.000595	−2.441665
H	1.774558	−0.180149	2.352424	C	−1.861113	1.887904	1.074503
C	3.333985	1.689516	−0.857874	H	−1.023681	2.473765	0.675294
H	2.765055	2.621029	−0.737294	H	−2.733313	2.552108	1.156295

H	4.346483	1.860322	−0.465607		H	−1.595663	1.544819	2.082495
H	3.416948	1.478996	−1.932241		Si	2.323802	0.241475	−0.031524
C	−3.390406	−1.065111	0.950836		C	2.335546	1.116775	1.612833
H	−4.176470	−0.652008	1.599132		H	1.782121	0.536107	2.360783
H	−3.866559	−1.758162	0.244376		H	3.367633	1.246812	1.968475
H	−2.690101	−1.634784	1.574003		H	1.880086	2.113483	1.537484
C	−3.639223	1.215243	−1.111843		C	3.123996	1.274667	−1.361159
H	−4.456455	1.697088	−0.556436		H	4.181939	1.454403	−1.122816
H	−3.096516	1.995597	−1.661430		H	3.077653	0.771040	−2.335595
H	−4.087106	0.531457	−1.845029		H	2.631347	2.251434	−1.457290
C	−1.637575	1.466945	1.219122		C	3.056816	−1.465143	0.063220
H	−1.047082	2.210750	0.669091		H	2.567128	−2.058639	0.845105
H	−2.379183	2.003065	1.828759		H	2.942700	−1.995901	−0.891049
H	−0.967459	0.921062	1.894188		H	4.130077	−1.405023	0.294278

$[(Me_3SiO)_3S]^+$ – C_1

E_0 = −1850.462627

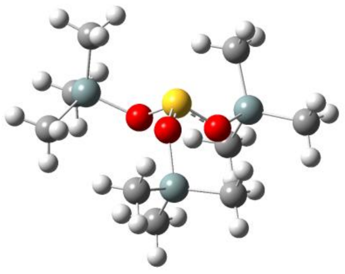

$[(Me_3SiO)_2S(O)SiMe_3]^+$ – C_1

E_0 = −1850.4314455

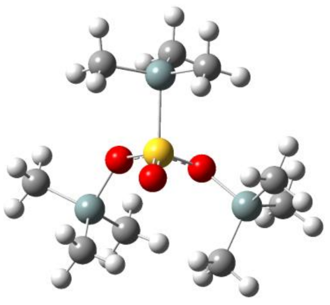

S	0.077565	−0.072262	−0.854703		S	−0.105548	0.470628	−0.343736
O	−0.825063	1.181574	−0.934586		O	−0.811740	−0.585082	0.557432
O	1.355701	0.479548	−0.138308		O	1.340730	0.524013	0.198597

O	−0.537404	−0.961914	0.286796		O	−0.194420	0.131047	−1.749758
Si	2.881812	−0.435067	0.095270		Si	−0.918418	2.560217	0.176179
Si	−1.837572	−2.172743	0.062873		C	−2.643160	2.489320	−0.497454
Si	−1.051992	2.617233	0.137076		H	−3.271200	1.773124	0.045142
C	−1.816959	−2.653300	−1.731329		H	−3.100150	3.483403	−0.382430
H	−2.574964	−3.431681	−1.899916		H	−2.653692	2.241016	−1.565944
H	−2.066914	−1.814023	−2.394363		C	0.254440	3.633062	−0.776452
H	−0.849510	−3.068502	−2.043107		H	−0.049123	4.683053	−0.651765
C	−3.395677	−1.302374	0.564326		H	1.283867	3.536594	−0.410929
H	−4.250170	−1.988247	0.477803		H	0.231662	3.405215	−1.849631
H	−3.351427	−0.961178	1.606619		C	−0.778271	2.629096	2.022560
H	−3.600057	−0.438613	−0.081860		H	0.263427	2.545157	2.355443
C	−1.321489	−3.522072	1.219651		H	−1.164653	3.598255	2.371381
H	−1.204218	−3.146400	2.244467		H	−1.371038	1.841241	2.503711
H	−2.089805	−4.307711	1.241486		Si	2.764317	−0.578393	0.085208
H	−0.378041	−3.988925	0.908666		C	2.514188	−1.831130	1.429505
C	2.846042	−1.823615	−1.140069		H	1.643666	−2.472265	1.243201
H	2.026646	−2.529990	−0.949711		H	3.397439	−2.482684	1.492015
H	2.769602	−1.461905	−2.173757		H	2.387022	−1.349054	2.407174
H	3.782884	−2.393623	−1.062640		C	4.138276	0.613747	0.427828
C	2.824886	−1.012596	1.854352		H	5.098355	0.078365	0.433389
H	3.749692	−1.552810	2.101542		H	4.198017	1.392352	−0.343414
H	2.739150	−0.167872	2.550066		H	4.022889	1.096541	1.406671
H	1.981781	−1.693052	2.029515		C	2.795691	−1.290649	−1.624024
C	4.169872	0.849013	−0.241464		H	1.949769	−1.961299	−1.815744
H	4.069451	1.704241	0.439555		H	2.793516	−0.505403	−2.390125

H	5.170704	0.421259	−0.087772		H	3.719457	−1.875014	−1.745468
H	4.118329	1.217472	−1.273906		Si	−1.844768	−1.982966	0.084236
C	0.328642	3.775488	−0.292453		C	−2.236214	−2.669659	1.758585
H	1.307369	3.373107	−0.004503		H	−2.883828	−3.551716	1.653977
H	0.342743	3.997980	−1.367216		H	−1.329085	−2.984105	2.290320
H	0.186582	4.726606	0.240513		H	−2.767622	−1.939304	2.381968
C	−2.715070	3.195249	−0.435170		C	−0.825052	−3.113304	−0.973701
H	−3.499209	2.458180	−0.219520		H	−1.418046	−4.010483	−1.203370
H	−2.983038	4.127011	0.083117		H	−0.554228	−2.640858	−1.925737
H	−2.718764	3.401740	−1.513112		H	0.087425	−3.450505	−0.465697
C	−1.030038	2.025934	1.895450		C	−3.318922	−1.295260	−0.805901
H	−1.796999	1.262491	2.075120		H	−3.901372	−0.613313	−0.173561
H	−0.054262	1.618566	2.185972		H	−3.038079	−0.777705	−1.731719
H	−1.247187	2.876108	2.558148		H	−3.983865	−2.125826	−1.083643

Table S5: Silylated isomers of $[SO_3]^{2-}$.

(We have also calculated the sulfite species for comparison)

	ΔG [kcal·mol^{-1}][a]	ΔΔG [kcal·mol^{-1}][b]
$[SO_3]^{2-}$	-	-
$[Me_3Si\text{-}SO_3]^-$	−274.93	
$[Me_3SiO\text{-}SO_2]^-$	−294.51	+19.57
$[Me_3Si\text{-}S(O)(OSiMe_3)]$	−130.79	
$[(Me_3SiO)_2\text{-}SO]$	−150.95	+20.16
$[Me_3Si\text{-}S(O)(OSiMe_3)_2]^+$	−28.08	
$[(Me_3SiO)_3\text{-}S]^+$	−48.67	+20.60

[a] for the reaction of $[Me_3Si]^+$ with the thermodynamically favored isomer in the gas phase; [b] energy difference between both isomers.

Table S6: Energies in kcal/mol.

Trimethylsilyl affinity (TMSA) of $A_{(g)}$ is defined as the negative of the reaction enthalpy $\Delta H_{(g)}°(298)$ in kcal/mol at 298.15 K for the reaction A(g) + T^+(g) → $[AT]^+_{(g)}$. The TMSA value given is for the conjugated acid−base pair $A_{(g)}/[AT]^+_{(g)}$.

**Base + T^+ = Base-T^+**	**ΔE**	**ΔH**	**ΔG**	**TMSA**
T_2SO_4	-57.79	-55.54	-43.96	55.54
DMAP	-79.98	-76.61	-62.40	76.61
$OPMe_3$	-75.40	-72.82	-60.10	72.82
T_2O	-45.48	-50.42	-16.44	50.42
T_2S	-53.94	-61.66	-25.52	61.66
$[SO_4]^{2-}$	-291.92	-215.89	-229.97	215.89
$[TSO_4]^-$	-159.26	-82.88	-93.90	82.88

T = Me_3Si

Table S7: Selected NBO data (partial charges in e)

species	atom		Partial charge
**$T_3SO_4^+$**	S		**2.702**
	O	Si	-1.016
	O	free	-0.875
	O	Si	-1.008
	O	Si	-1.019
		**sum**	**-1.216**
		**qCT**	**0.784**
		qCT/Si group	0.261
	Si		1.935
	Si		1.938
	Si		1.937
	**avarage**		**1.937**
**T_2SO_4**	S		**2.639**
	O	free	-0.935
	O	free	-0.935
	O	Si	-1.059
	O	Si	-1.059
		**sum**	**-1.350**
		**qCT**	**0.650**
		qCT/Si group	0.325
	Si		1.950
	Si		1.950
	**avarage**		**1.9498**
**TSO_4^-**	S		**2.589**
	O		-1.004
	O		-1.037
	O		-1.009
	O		-1.121
		**sum**	**-1.582**
		**qCT**	**0.418**
		qCT/Si group	0.418
	Si		1.972
	**avarage**		**1.972**

T = Me_3Si

Table S8: Selected calculated and (*observed*) NMR shifts of $[NSO]^-$, H-NSO, Me_3Si-NSO and $B(C_6F_5)_3$, $GaCl_3$ and $[Me_3Si]^+$.

	$\delta^{17}O$ [ppm]	$\delta_{calc.}{}^{17}O$ [ppm]
$[H_3O]^+$	-	14.7
$[SO_4]^{2-}$	-	215.8
$K_2[SO_4]$	167.1	
$[HSO_4]^-$	*160.0 [KHSO₄]*	222.7 (SO)<br>210.8 (OH)
H_2SO_4	151.88	218.5 (SO)<br>202.5 (OH)
$[SO_3]^{2-}$		317.3
$[SO_2(OH)]^-$		300.1 (SO)<br>267.6 (OH)
$[HSO_3]^-$		247.1
$OS(OH)_2$		251.9 (SO)<br>227.8 (OH)
$HSO_2(OH)$		237.6 (SO)<br>207.8 (OH)
$[NH_4]_2[SO_4]$	168.3	
$(Me_3SiO)_2SO_2$	152.6 (OSi)<br>174.5 (OS)	215.6 (OSi)<br>232.2 (OS)
$[DMAP\text{-}SiMe_3]_2[SO_4]$	169.0	
$[DMAP\text{-}SiMe_3][Me_3SiO\text{-}SO_3]$	167.8	219.9 (OSi)<br>234.3 (OS)
$Na_2[SO_3]$	210.4	
$Ag[O_3SCF_3]$	158.9	216.2
$K_2[S_2O_7]$	*160.2 decomposition*	226.2 (SO)<br>332.5 (SOS)
$Na_2[S_2O_3]$	227.4	302.9

7 References

[1] Purification of Laboratory Chemicals, 5. Ed. W. L. F. Armarego, **2003**.

[2] T. J. Curphey, *Phosphorus Sulfur Silicon Relat. Elem.* **2001**, *173*, 123–142.

[3] M. G. Voronkov, V. K. Roman, E. A. Maletina, *Synthesis (Stuttg).* **1982**, *1982*, 277–280.

[4] M. Lehmann, A. Schulz, A. Villinger, *Angew. Chem. Int. Ed.* **2009**, *48*, 7444–7447.

[5] M. Lehmann, A. Schulz, A. Villinger, *Angew. Chem.* **2009**, *121*, 7580–7583.

[6] C. A. Reed, *Acc. Chem. Res.* **2010**, *43*, 121–128.

[7] A. Franken, B. T. King, J. Rudolph, P. Rao, B. C. Noll, J. Michl, *Collect. Czechoslov. Chem. Commun.* **2001**, *66*, 1238–1249.

[8] Z. Xie, T. Jelínek, R. Bau, C. A. Reed, *J. Am. Chem. Soc.* **1994**, *116*, 1907–1913.

[9] G. B. Dunks, K. Barker, E. Hedaya, C. Hefner, K. Palmer-Ordonez, P. Remec, *Inorg. Chem.* **1981**, *20*, 1692–1697.

[10] G. B. Dunks, K. Palmer-Ordonez, *Inorg. Chem.* **1978**, *17*, 1514–1516.

[11] T. S. Cameron, A. Decken, I. Krossing, J. Passmore, J. M. Rautiainen, X. Wang, X. Zeng, *Inorg. Chem.* **2013**, *52*, 3113–3126.

[12] G. Bauer, H. Mikosch, *J. Mol. Struct.* **1986**, *142*, 21–24.

[13] J. Goubeau, W. Bereger, *Z. anorg. allg. Chem.* **1960**, *304*, 147–153.

[14] G. M. Sheldrick, *Acta Cryst. A* **2015**, *71*, 3–8.

[15] G. M. Sheldrick, *Acta Cryst. C* **2015**, *71*, 3–8.

[16] G. M. Sheldrick, *SADABS Version 2*, University of Göttingen, Germany, **2004**.

[17] *Gaussian 09, Revision E.01*, M. J. Frisch, G. W. Trucks, H. B. Schlegel, G. E. Scuseria, M. A. Robb, J. R. Cheeseman, G. Scalmani, V. Barone, B. Mennucci, G. A. Petersson, H. Nakatsuji, M. Caricato, X. Li, H. P. Hratchian, A. F. Izmaylov, J. Bloino, G. Zheng, J. L. Sonnenberg, M. Hada, M. Ehara, K. Toyota, R. Fukuda, J. Hasegawa, M. Ishida, T. Nakajima, Y. Honda, O. Kitao, H. Nakai, T.

Vreven, J. A. Montgomery Jr., J. E. Peralta, F. Ogliaro, M. Bearpark, J. J. Heyd, E. Brothers, K. N. Kudin, V. N. Staroverov, T. Keith, R. Kobayashi, J. Normand, K. Raghavachari, A. Rendell, J. C. Burant, S. S. Iyengar, J. Tomasi, M. Cossi, N. Rega, J. M. Millam, M. Klene, J. E. Knox, J. B. Cross, V. Bakken, C. Adamo, J. Jaramillo, R. Gomperts, R. E. Stratmann, O. Yazyev, A. J. Austin, R. Cammi, C. Pomelli, J. W. Ochterski, R. L. Martin, K. Morokuma, V. G. Zakrzewski, G. A. Voth, P. Salvador, J. J. Dannenberg, S. Dapprich, A. D. Daniels, O. Farkas, J. B. Foresman, J. V. Ortiz, J. Cioslowski, D. J. Fox, Gaussian, Inc., Wallingford CT, **2013**.

[18] E. D. Glendening, J. K. Badenhoop, A. E. Reed, J. E. Carpenter, J. A. Bohmann, C. M. Morales, C. R. Landis, F. Weinhold, **2013**.

[19] J. E. Carpenter, F. Weinhold, *J. Mol. Struct.: THEOCHEM* **1988**, *169*, 41–62.

[20] F. Weinhold, J. E. Carpenter, *The Structure of Small Molecules and Ions*, Plenum Press, **1988**.

[21] F. Weinhold, C. R. Landis, *Valency and Bonding. A Natural Bond Orbital Donor-Acceptor Perspective*, Cambridge University Press, **2005**.

[22] J. P. Perdew, K. Burke, M. Ernzerhof, *Phys. Rev. Lett.* **1996**, *77*, 3865–3868.

[23] J. P. Perdew, K. Burke, M. Ernzerhof, *Phys. Rev. Lett.* **1997**, *78*, 1396–1396.

[24] M. Ernzerhof, G. E. Scuseria, *J. Chem. Phys.* **1999**, 110, 5029−5036.

[25] C. Adamo, V. Barone, *J. Chem. Phys.* **1999**, *110*, 6158–6170.

[26] T. H. Dunning Jr., *J. Chem. Phys.* **1989**, *90*, 1007–1023.

[27] T. H. Dunning Jr., D. E. Woon, *J. Chem. Phys.* **1993**, *98*, 1358–1371.

[28] K. A. Peterson, T. H. Dunning Jr., *J. Chem. Phys.* **2002**, *117*, 10548–10560.

[29] E. D. Glendening, A. E. Reed, J. E. Carpenter, F. Weinhold, in *NBO Version 3.1*, **n.d.**

[30] J. E. Carpenter, F. Weinhold, *J. Mol. Struct. (Theochem)* **1988**, *169*, 41–62.

[31] J. E. Carpenter, F. Weinhold, *The Structure of Small Molecules and Ions*, Plenum Press, New York (United States of America), **1988**.

[32] F. Weinhold, C. R. Landis, *Valency and Bonding. A Natural Bond Orbital Donor-Acceptor Perspective*, Cambridge University Press, Cambridge (United

Kingdom), **2005**.

[33] F. London, *J. Phys. Radium* **1937**, *8*, 397–409.

[34] R. McWeeny, *Phys. Rev.* **1962**, *126*, 1028–1034.

[35] R. Ditchfield, *Mol. Phys.* **1974**, *27*, 789–807.

[36] K. Wolinski, J. F. Hinton, P. Pulay, *J. Am. Chem. Soc.* **1990**, *112*, 8251–8260.

[37] J. R. Cheeseman, G. W. Trucks, T. A. Keith, M. J. Frisch, *J. Chem. Phys.* **1996**, *104*, 5497–5509.
